# Evaluating the ecological and social targeting of a compensation scheme in Bangladesh

**DOI:** 10.1371/journal.pone.0197809

**Published:** 2018-06-13

**Authors:** Annabelle Jade Bladon, Essam Yassin Mohammed, Belayet Hossain, Golam Kibria, Liaquat Ali, E. J. Milner-Gulland

**Affiliations:** 1 Imperial College London, Silwood Park Campus, Ascot, Berkshire, United Kingdom; 2 International Institute for Environment and Development, London, United Kingdom; 3 Bangladesh Centre for Advanced Studies, Dhaka, Bangladesh; 4 Department of Zoology, University of Oxford, Oxford, United Kingdom; Public Library of Science, UNITED KINGDOM

## Abstract

Conservation payments are increasingly advocated as a way to meet both social and ecological objectives, particularly in developing countries, but these payments often fail to reach the ‘right’ individuals. The Government of Bangladesh runs a food compensation scheme that aims to contribute to hilsa (*Tenualosa ilisha*) conservation by improving the socioeconomic situation of households affected by hilsa sanctuary fishing bans. Analysing data from a household survey of compensation recipients and non-recipients, we identify the current correlates of compensation distribution and explore perceptions of fairness in this distribution. We find that distribution is largely spatial rather than based on the household characteristics that are supposed to determine eligibility for compensation, indicating political influence in the distribution process. We also find the compensation scheme is widely perceived to be unfair, which could be undermining its potential to compensate vulnerable fishers while improving compliance with fishing bans. The spatial distribution of compensation would shift substantially under alternative targeting scenarios that are likely to improve the cost-effectiveness of the scheme, such as targeting those who are most dependent on fishing for their livelihood. This study highlights a challenge for conservation payment schemes that aim to achieve the dual objectives of poverty reduction and ecological sustainability, particularly large-scale public schemes, and suggests that more effective targeting and transparency about the basis of payment distribution are prerequisites for schemes to be both cost-effective and socially acceptable.

## Introduction

Monetary or in-kind payments are increasingly used in resource management and conservation as a way to incentivise behavioural change or compensate for losses incurred as a result of intervention [1–3]. Compensation payments aim to offset conservation costs, whereas incentive payments aim to change behaviour voluntarily and may have additional benefits; a conservation payment scheme may have one or both of these objectives. The type of conservation payment that has received most attention in the literature is Payments for Ecosystem Services (PES), a subset of incentive-based approaches offering conditional positive incentives for behavioural change [4–6]. However, more straightforward compensation payments are also widespread. Conservation payments are widely advocated for their potential to meet both social and ecological objectives, especially in developing countries [2,7–11]. Cost-effective conservation payments must target the ‘right’ individuals to attain maximum social-ecological additionality with the available financial resources (i.e., those individuals, the targeting of whom has the potential to deliver the best social-ecological outcomes, compared to what would have occurred without the intervention). This study focuses on targeting as the first element required for cost-effectiveness.

Conservation payments may have an implicit or explicit social side objective, with government-financed schemes often using measures of poverty and vulnerability as specific targeting criteria [12]. Vulnerability, a crucial component of poverty, is generally defined as the degree to which a system or individual is susceptible to and unable to cope with adverse effects of a stress or change [13]. As a forward-looking component, it is thus thought to play a central role in poverty reduction [14]. A key determinant of vulnerability is dependence; households which are less dependent on one occupation or resource are likely to be less sensitive to, and more able to cope with, stress affecting that occupation or resource [15]. Minimising the negative impacts of an intervention, or maximising its positive impacts, for the most vulnerable or dependent groups should not only enable equitable social impacts, but also improve perceptions of the fairness of that intervention. These perceptions can in turn promote community acceptance, compliance with regulations and thereby enhance the conservation impact of an intervention [16,17].

Even when interventions are specifically targeted for social objectives, benefits may still fail to reach the ‘right’ individuals [9]. For instance, compensation intended to safeguard vulnerable households in Madagascar, who were negatively affected by an intervention under the climate mechanism REDD+, did not reach the most vulnerable groups due to a combination of elite capture and systematic bias in the assessment process [2]. There is a great deal of literature from development that highlights the risk of ineffective social targeting through inclusion or exclusion errors and elite capture of benefits [18–20]. Despite the familiarity of the problem of elite capture in conservation [17,21], the targeting of poverty-focused conservation payments has received limited attention (with one notable exception [2]).

In this paper, we focus on a government-led compensation scheme for hilsa (*Tenualosa ilisha*) fishing communities in Bangladesh. The hilsa fishery is a small-scale coastal marine and freshwater fishery, which is largely artisanal and supports the livelihoods of up to 500,000 people [22]. In response to reported stock declines, since 2003 the government has introduced various regulations for the protection of jatka (juvenile hilsa up to 25 cm in length): a) jatka fishing and related activities are banned from November to July across the country; b) monofilament gillnets are banned; and c) five hilsa sanctuaries are closed to fishing for two months of each year [23]. In recognition of the socioeconomic hardships imposed by these regulations, the government in 2004 started piloting a food grain compensation scheme, distributing rice and wheat to fishers living inside and around the sanctuary areas, during the perceived peak period of jatka presence (February to May). Funded through the pre-existing national Vulnerable Group Feeding (VGF) programme, which aims to reduce food insecurity [24,25], this compensation is intended to reduce the vulnerability of affected fishers [26]. Although it does not have the conditionality that defines a PES, the scheme is expected to incentivise compliance with the jatka fishing regulations, which are poorly enforced [27,28]. The amounts of compensation available are largely determined by the availability of VGF resources, but allocations have increased over time in response to needs assessments [26]. When the scheme formally began in 2008, 145,335 fishers were allocated 10 kg of wheat per household for one to three months of the year, dependent on location, and by 2014, 224,102 households were allocated 40 kg of rice per household for four months of the year (S1 Table). In 2008, the government also introduced some alternative income generation support for hilsa fishers, but coverage of this support has declined [23] and we do not include it in our analysis.

Since resources are limited, the compensation scheme is not open to all fishers who are affected by hilsa regulations, but directed towards the ‘poorest and most vulnerable’ of the affected fishers [29]. There are no prescribed selection criteria, but the government’s Department of Fisheries (DoF) claims to target ‘real jatka fishers’, those who are ‘fully dependent’ on fishing for their livelihoods, and those without assets such as agricultural land or boats (M. Mome, DoF, personal communication, 1/9/2014). Each local council is invited to put forward a list of jatka fishers, which is finalised through a complex process at various levels of government [26]. However, concerns have been raised regarding political interference in this process and the distribution of compensation, and thus its equitability [23,26,28]. Indeed, social safety net schemes in Bangladesh, including the VGF, tend to be characterised by high levels of inclusion and exclusion error and elite capture [30–32]. A recent assessment of jatka fisher ‘rehabilitation’ approaches identified some issues in the compensation distribution process and made recommendations for improvements, but sheds little light on whether those who are getting the payments are the most eligible, based on the stated objectives of the scheme [33].

We therefore use a household survey to examine the targeting of the compensation scheme for hilsa conservation in Bangladesh. We first quantify and compare relative household fishing dependence, explore the components of this dependence, and investigate the association between receipt of compensation and compliance with regulations, before investigating the correlates of compensation distribution. Expectations for these correlates were based on the officially reported rationale behind the scheme (Table 1). As this is a post-hoc study, we focus not on whether the scheme was fit for purpose at its inception, but rather on who its current recipients are, and how the spatial distribution would change from its current state if recipients were chosen based on prescribed scheme criteria. We also assess the perceived sufficiency of compensation provided. Finally, we investigate the perceptions of fairness and legitimacy of compensation. This allows us to evaluate whether and how the scheme could be redesigned to more effectively fulfil its goals.

**Table 1:** Summary of hypothesised correlates of the probability of receiving compensation.

Correlate	Hypothesis	Explanation
Fishing dependence	Fully dependent fishers are more likely to receive compensation.	The scheme is officially aimed at fully dependent fishers.
Jatka fishing	Jatka fishers are more likely to receive compensation.	The scheme is officially aimed at jatka fishers.
Income	Low income households are more likely to receive compensation.	Low income is an indicator of poverty and vulnerability [34].
Debt	Households who have taken loans are more likely to receive compensation.	Loan taking is a typical coping strategy of the poorest fishers in Bangladesh and an indicator of vulnerability [33,35].
Food insecurity	Households who consume less or cheaper food as a coping strategy during ban periods are more likely to receive compensation.	Households who consume less or cheaper food as a coping strategy during ban periods suffer from food insecurity, which is a dimension of poverty [36].
Household size	Larger households are more or less likely to receive compensation.	Large households may be more or less vulnerable depending on the balance between production and consumption [37–39].
Household dependency ratio	Households with a high proportion of dependents are more likely to receive compensation.	A high dependency ratio increases vulnerability [39].
Fisher association membership	Members are more likely to receive compensation.	Members have more social capital and influence [40–42].
Sanctuary area	Households inside sanctuary areas are more likely to receive compensation.	The scheme is officially aimed at fishers living inside sanctuaries because they experience a complete fishing ban and so are likely to lose the most earnings.
District	Fishers in some districts may be more likely to receive compensation.	Geographic clustering of targeting [28].
Village	Fishers in some villages may be more likely to receive compensation.	Geographic clustering of targeting [28].

## Materials and methods

### Data collection

**Fig 1:**
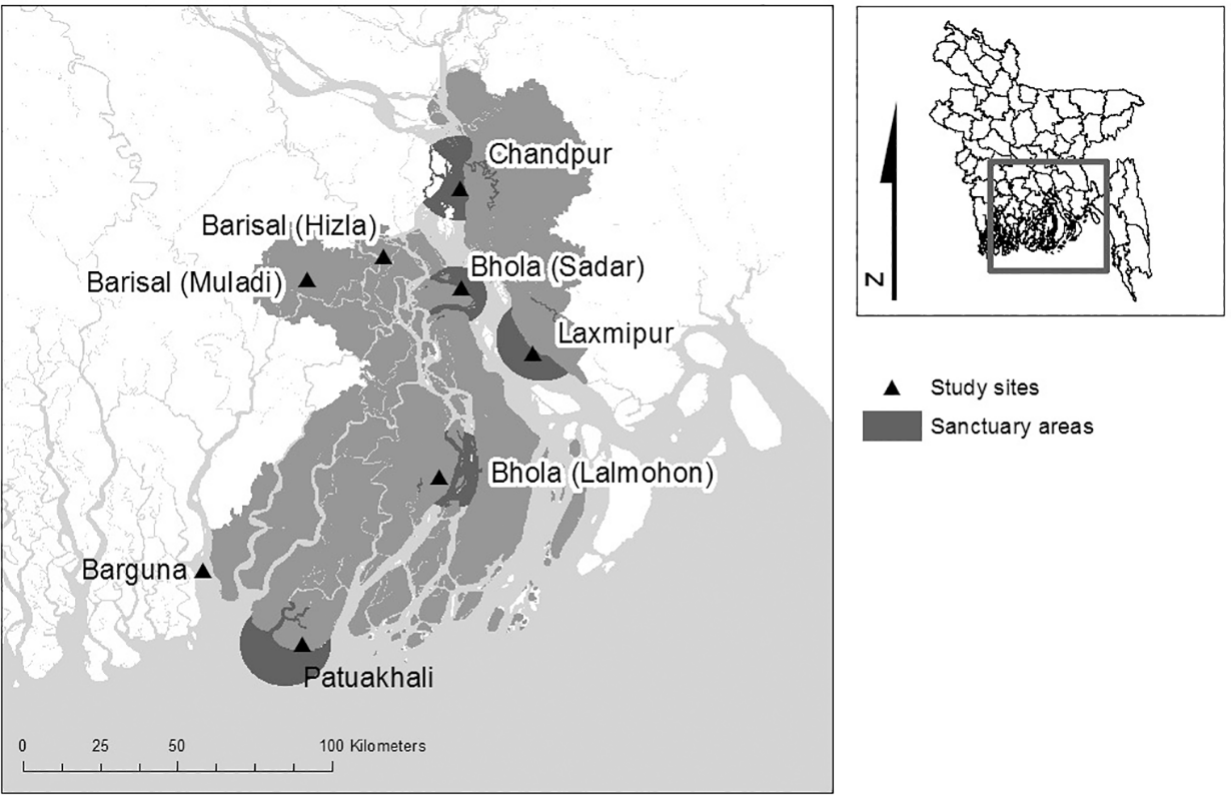
Map of study area, showing study site districts in relation to sanctuary sites. Each study site represents the approximate location of a cluster of surveyed villages, denoted by the relevant district name (precise village coordinates were not available). In Barisal and Bhola districts two village clusters were sampled and can be distinguished by the sub-district names (in brackets); in the other districts just one village cluster was sampled.

We interviewed 800 households between May and October 2014 in the lower Meghna River region where the compensation scheme operates (Fig 1). We developed the questionnaire (S1 Appendix) for a larger survey on the basis of focus group discussions previously carried out in five sites from October to December, 2013. We conducted a pilot survey with 28 households in April 2014, feedback and observations from which allowed the questionnaire to be refined, and we made efforts to identify and minimise sources of bias [43]. We followed the ethical principles of the International Institute for Environment and Development, developed by the Research Quality Group [44]. This involved obtaining free, prior and informed oral consent from all participants. Written consent was not obtained due to low literacy rates in the study area and, given that we were asking about potentially illegal and sensitive behaviour, it was not appropriate for us to document individual consent to participate. Data were analyzed anonymously for the same reason. IIED oversees ethics through its Research Quality Group, but it does not have a specific Institutional Review Board. The use of oral consent was thus not officially approved by a committee, but it clearly abides by IIED’s agreed research ethics frameworks [44].

We selected survey households from 19 villages across six districts through stratified random sampling (S2 Appendix). 600 of the households lived in hilsa sanctuary areas (Chandpur, Laxmipur, Bhola, Patuakhali districts) and 200 lived in districts outside sanctuary areas (Barisal and Barguna; Fig 1). In the sanctuary areas, 150 households were sampled from each district, but due to resource constraints, district sample sizes were smaller outside sanctuary areas (125 households in Barisal district and 75 in Barguna district). We aimed to balance the proportions of recipients and non-recipients interviewed within the compensation areas; 54% of compensation area households were recipients and 46% were non-recipients (S2 Appendix).

Recent estimates of the total number of hilsa fishers in Bangladesh range from 300,000 (A. Wahab, WorldFish, personal communication, 21/03/2014) to 500,000 (M. Mome, Department of Fisheries, personal communication, 20/03/2015) and according to M. Mome, 224,102 households received compensation in 2014, which is around 45–75% of households affected by the hilsa conservation regulations, or 65%, according to a recent study [33]. In the study sites 60% of households were officially reported to have received compensation, and 54% of surveyed households said they received compensation, indicating that the sample is roughly representative of the recipient and non-recipient groups (S2 Appendix). 632 respondents were household heads and 126 respondents were women, of whom 125 were not household heads.

### Data analysis

#### Compensation distribution

In order to identify the correlates of compensation distribution, we fitted binomial generalised linear mixed effects models (GLMMs) with the probability of receiving compensation as a binary response variable (1 = compensation recipient, 0 = non-recipient). We fitted the GLMMs as random intercept models with district and village as grouping factors in the random effects and a probit link function. Models were fitted in R version 3.4.3 [45] using the package lme4 [46]. We selected the best random effects structures using likelihood ratio tests and validation plots [47], and estimated the models using maximum likelihood with the Laplace approximation. A summary and description of the explanatory variables can be found in Table 2.

**Table 2:** List, type and description of explanatory variables investigated through GLMMs.

Explanatory variables	Type	Description	Expected influence^a^	
			Model (a)	Model (b)
**Fixed effects**				
Sanctuary area	Binary	Households may live within a sanctuary (1) or outside a sanctuary (0)	+	+/-
Jatka fishing	Binary	Fishers may target jatka (1) or not (0), based on survey question 23 (S1 Appendix)	+	+/-
Compensation	Binary	Households may receive compensation (1) or not (0), based on survey question 28a (S1 Appendix)		+
Fishing dependence	Continuous	Index measuring household dependence on fishing, based on methods presented in S3 Appendix	+	+/-
Respondent identity^b^	Binary	Household head (1) or other (0), based on survey question 1 (S1 Appendix)	+/-	+/-
Awareness	Binary	Aware of all management interventions (1) or not (0), based on survey question 27 (S1 Appendix)		+
Fisher association membership	Binary	Fishers may be members of associations (1) or not (0), based on survey question 20 (S1 Appendix)	+	+
Household size	Continuous	Number of household members, based on survey question 5 (S1 Appendix)	+	
Household dependency ratio	Continuous	Household dependency ratio (number of economic earners/non-earners), based on survey questions 5 and 7 (S1 Appendix)	+	
Food insecurity	Binary	Households may use food-based coping strategies during fishing ban (1) or not (0), based on survey question 42 (S1 Appendix)	+	
Debt	Binary	Households may have taken a loan (1) or not (0), based on survey question 42 (S1 Appendix)	+	
Household income	Continuous	Monthly income per capita in BDT (average household monthly income from fishing + (annual income from other sources/12)/household size), based on survey questions 5, 17 and 18 (S1 Appendix)	-	+
**Random effects**				
District	Categorical	6 level factor		
Village	Categorical	19 level factor		

Model (a) was for the probability of receiving compensation; and (b) was for the probability of perceiving fair compensation distribution.

^a^ Blanks indicate where fixed effects were not included in models.

^b^ We included whether or not the respondent was the household head to account for confounding variables, since respondent identity was highly correlated with age, gender, and years of education, which might in turn be expected to influence compensation distribution and perceptions of fairness among household-head respondents.

Since fishing dependence is a multidimensional concept, we used factor analysis for mixed data (FAMD) in the R package FactoMineR [48,49] to develop an index of fishing dependence, aggregating a range of variables known from the literature to play a meaningful role in fishing dependence (S3 Appendix). FAMD is a principal component method that can balance the influence of continuous and categorical variables [50,51]. Following the methods of [52], we carried out descriptive analyses to inform final variable selection for the FAMD, only using variables that were significantly correlated (*p* < 0.05) with the majority of the others (S3 Appendix). We selected the first dimension as a multivariate indicator of dependence, as is the convention in the construction of socioeconomic indices [52,53]. The index ranged from -3.44 to 2.63, so we rescaled it (-1 to +1) for more intuitive interpretation.

We explored collinearity among explanatory variables using pairwise plots, Spearman’s rank correlation coefficient, and phi coefficient. Most of the variables lacked significant correlations (*p* > 0.05) and those identified were weak (-0.5 > *ɸ* or *r*_*s*_ < 0.5, *p* < 0.05). We followed an information-theoretic approach to model selection [47,54]. We fitted all possible combinations of explanatory variables using Maximum Likelihood (ML) estimation procedures with the R package MuMIn [55], and selected top candidate models according to the corrected Akaike Information Criterion (AICc). No models were clearly superior (weights of top models were < 0.9), so we re-ran those with **Δ**AICc < 4 using Restricted Maximum Likelihood (REML) estimation procedures for accurate parameter estimates [56], which we then averaged across these models, allowing relative variable importance to be determined [57]. We presented coefficients for the full average, rather than the subset or conditional average, which has a tendency of biasing the values away from zero [58]. We standardised continuous explanatory variables were standardised by two standard deviations for direct comparison of coefficients following model averaging [57,59].

We checked models for residual normality, heteroskedasticity and correlations between fixed effects and the residuals. Eight households had missing data and were excluded from analysis. To analyse spatial effects on the probability of receiving compensation, we estimated best linear unbiased predictors (BLUPs) from the global models, which measured the residual effect associated with each random effect (district and each village within district).

To explore how the spatial distribution of compensation would change under potential alternative targeting scenarios, we calculated the current proportions of households receiving compensation in each district against the proportions that would receive compensation if a) all jatka fishers were targeted and b) if the most fishing dependent households were targeted. Budget restrictions meant that 60% of households in the study area were reportedly compensated in the year of study (S2 Appendix), so we selected a) all jatka fishers (53% of total respondents); and b) the top 60% of households, in order of their fishing dependence.

#### Fairness of compensation distribution

Perceived fairness is a critical determinant of the acceptability of benefit distribution [17,60]. We asked respondents whether they perceived the distribution of compensation to be fair (yes or no). In order to explore these perceptions of fairness or unfairness, we followed the above methods to fit binomial random intercept GLMMs with the probability of perceiving fairness as a binary response variable (1 = fair, 0 = unfair). Explanatory variables and expectations are presented in Table 2. We expected households with a high level of awareness of hilsa management interventions (defined as ‘aware of all three interventions discussed in the questionnaire’) to be more likely to feel the scheme was fair, assuming that their understanding of the rationale behind compensation distribution was also greater [61]. Research on fairness in payment systems has found it to depend on the quality of local governance and to be higher among local association members [17]. We therefore expected fisher association members to perceive fair distribution, and to see variation in perceptions between villages and between districts due to differences in local governance. We expected compensation recipients to perceive fair distribution, but expectations for high fishing dependence, jatka fishers and sanctuary fishers were less clear.

## Results

### Fishing dependence and compliance with regulations

99.7% of respondents reported hilsa fishing to be their main income-generating activity, but local knowledge indicates that the proportion is nearer 70% (BH, personal observation). It is possible that some respondents overstated their involvement in hilsa fishing because of their awareness of the compensation scheme and purpose of the survey, but it could also be due to different interpretations of the term ‘main’ activity (e.g., as more than half of all income, or largest single source of income). 53% of respondents said that they target jatka, and there was no significant association between targeting jatka and receiving compensation (*χ*^2^ = 0.01; df = 1; *p* = 0.91). In fact, 52% of non-recipients said they target jatka, compared to 53% of recipients). 76% had livelihoods other than fishing, though only 8% had more than one alternative (S4 Appendix). The mean proportion of income from fishing was 82% (estimated based on reported average monthly income from fishing and reported annual income from other sources, see questions 17 and 18, S1 Appendix), showing a widespread high level of income dependency.

40% of respondents stated that their main coping strategy during fishing bans is to fish anyway, rather than to take another job or adopt a food-based or monetary coping strategy. It is not possible to conclude from this result why they keep fishing, but, since this particular response was not offered as an option by the enumerators, the high level of response supports its importance, and so justifies its use as an indicator of dependence. This result also indicates widespread non-compliance with the fishing bans.

When hilsa fishers were asked directly about compliance with fishing bans, the majority said that few fishers comply and none said that all fishers comply. Levels of compliance by compensation recipients were perceived to be significantly higher than those by non-recipients; 66% of respondents said that few or no recipients comply, while 78% said that few or no non-recipients comply (χ^2^ = 26.5, df = 3, *p* < 0.001). Supporting this perception, there was a significant negative association between fishing as a main coping strategy during the fishing bans and receiving compensation; 36% of compensation recipients said that they fish anyway as their main coping strategy during fishing bans, compared to 45% of non-recipients (*χ*^2^ = 8.06; df = 1; *p* < 0.01). This could indicate that people who fish during the ban periods are less likely to be compensated, but, given the poor monitoring and enforcement it is more likely an indication that people who are compensated are less likely to fish during the ban periods.

Households who said they fished in ban periods had significantly higher proportions of income from fishing than others (Wilcoxon rank sum test *W* = 31244.5; *p* < 0.001) and there was a significant negative association between having other livelihoods and fishing as a main coping strategy (*χ*^2^ = 129.54; df = 1; *p* < 0.001). This indicates that households tend to fish as a coping strategy because they have few or no other livelihoods, rather than that households tend to seek other livelihoods so that they don’t have to fish. Fishing has long been a way of life for Hindus in coastal areas of Bangladesh, and now there are increasing numbers of poor and landless Muslims engaging in fishing [62].

The index of fishing dependence primarily contrasted households with a high dependence on fishing (who own boats, use multiple fishing gears, fish illegally, have higher proportions of income from fishing, and have no agricultural land or other livelihoods) with households who are less dependent on fishing (who have agricultural land and other livelihoods, do not own boats, use a single gear type, do not fish illegally, and have lower proportions of income from fishing) [S3 Appendix].

### Compensation distribution

99.6% of households officially listed as compensation recipients also said during the survey that they received compensation. All of the reasons given for not receiving compensation by the small number of respondents officially listed as recipients, but who said that they did not receive compensation, fell under the umbrella of corruption by local Government officials, a widely cited problem in Bangladesh’s small-scale fisheries [23,26,63]. Of those who did receive it, 99% said they generated some income from fishing, while 96% said they fished for hilsa.

The most important fixed effect for the probability of receiving compensation was household size, which had a positive effect and a relative importance of 0.85 (larger households were more likely to receive compensation; Table 3A), though support for the model was weak (S2 Table). Fisher association membership also had some support for inclusion in top models (relative importance 0.68) but, contrary to expectations, households involved with fisher associations were less likely to receive compensation. Food insecurity had some support for inclusion in top models (relative importance 0.50), with a positive effect on the probability of receiving compensation, as expected. The other fixed effects had relative importance values of < 0.5 and received very little support for inclusion in the top models.

**Table 3:** Result for GLMMs of probability of (a) receiving compensation; and (b) perceiving fair compensation distribution.

	1. Probability of receiving compensation		2. Probability of perceiving fair distribution	
Fixed effects^a^	Estimate^b^ (SE)	Relative importance^c^	Estimate^b^ (SE)	Relative importance^c^
Intercept	-0.38 (0.50)		-11.10 (725.00)	
Compensation (1 = yes, 0 = no)			21.10 (1589.00)	1.00
Household size	0.25 (0.12)	0.85		
Fisher association membership (1 = yes, 0 = no)	-0.50 (0.29)	0.68	+	0.25
Food insecurity (1 = insecure, 0 = secure)	0.26 (0.18)	0.50		
Household dependency ratio	-	0.43		
Household income (BDT)	+	0.41	-	0.17
Respondent identity (1 = household head, 0 = other)	+	0.15	-	0.19
Jatka fishing (1 = yes, 0 = no)	+	0.12	-	0.25
Index of fishing dependence	-	0.12	-0.40 (0.35)	0.73
Loan (1 = yes, 0 = no)	-	0.12		
Inside sanctuary (1 = yes, 0 = no)	-	0.12	+	0.49
Awareness (1 = high, 0 = low)			+	0.17
**# of models in candidate set**	87		45	
**Random effects**^d^				
Village	0.37 [0.61]		3.04 [1.74]	
District	1.27 [1.13]		0.42 [0.65]	

Showing the model-averaged coefficient estimates (SE) and relative importance of each variable from the candidate set of models where ΔAICc < 4, based on 792 households from 19 villages in 6 districts.

^a^ Blanks indicate where fixed effects were not included in models.

^b^ Coefficient estimates are presented as contrasts from the intercept, standardised on two standard deviations following [59]. The directions of coefficient estimates were 100% consistent between model runs, excluding those for ‘Loan’ (one run was + and the other -).

^c^ Where the relative importance of a variable is < 0.5, only the direction of the effect is presented.

^d^ Random effects estimates of variance [SD] were taken from the global model.

There was clear spatial variation in the probability of receiving compensation, and the estimates of the random effects, particularly district, were larger than any of the fixed effects. The BLUPs for each district and for each village within district illustrate this spatial effect (S1 Fig). Households in Chandpur district were significantly more likely to receive compensation, while households with the same level of fishing dependence and other demographic characteristics in Bhola and Patuakhali were less likely to receive it.

**Fig 2:**
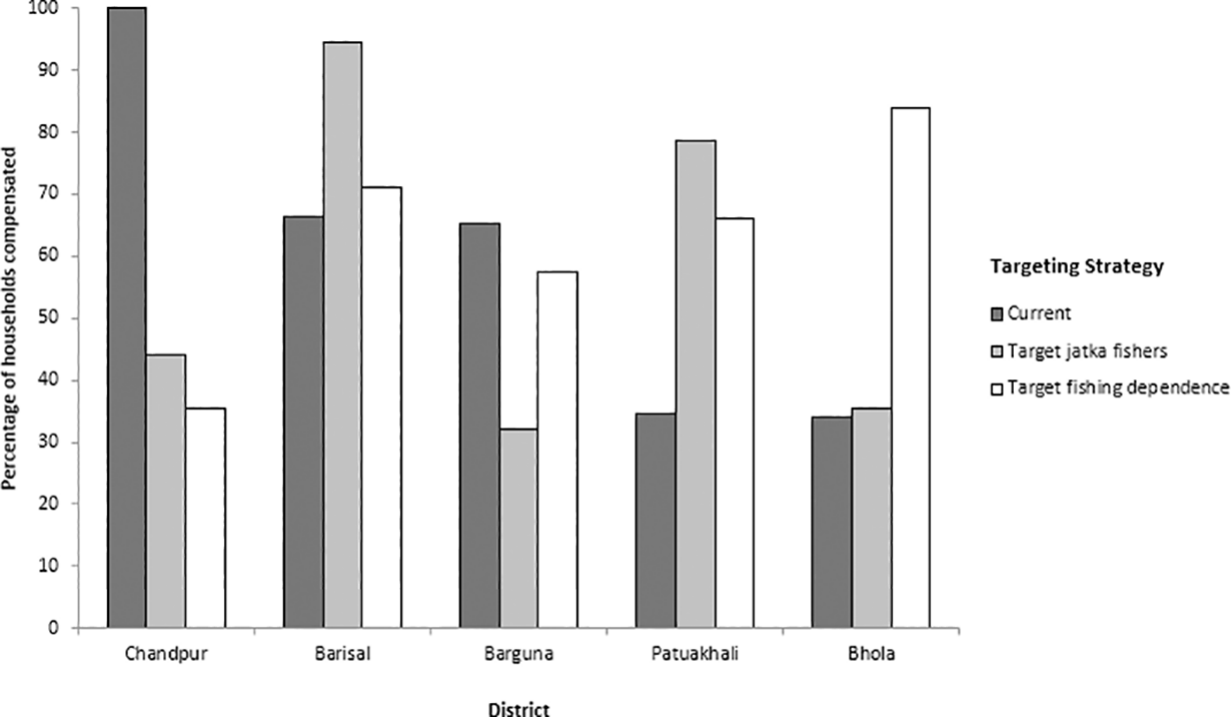
The percentage of study households compensated in each district under current and alternative targeting scenarios. The alternative scenarios presented are: targeting jatka fishers (52%) and targeting high fishing dependence (60%).

The highest coverage of fishing households by the compensation scheme is in the districts of Barisal, Barguna and, in particular, Chandpur, where 100% of study households said they receive compensation (Fig 2). If the 60% of households most dependent on fishing were targeted, coverage in Bhola, Patuakhali, Laxmipur and Barisal would increase (by 50%, 31%, 11% and 5%, respectively) at the expense of Chandpur and Barguna, where coverage would drop by 65% and 11% respectively. Similarly, if only jatka fishers were targeted (53% of households), Chandpur coverage would drop by 56% and Barguna by 33%, while Patuakhali coverage would increase by 44% and Barisal by 2%. This indicates that if the scheme were to be targeted more carefully according to its stated goals, there would be a shift in focus from Chandpur and Barguna to other districts, most noticeably to Patuakhali and Barisal.

Given the 100% coverage of households in Chandpur, we re-ran these models excluding households from Chandpur (S3 Table). Spatial variation in the probability of receiving compensation was substantially reduced, and the effect of district was almost 0 (S4 Table). Fixed effects remained similar to previous model runs, except for sanctuary area, which had a relative importance of 1 and a much stronger negative effect).

### Fairness and legitimacy of compensation distribution

60% of compensation recipients said that the compensation provided was insufficient, 39% said it was sufficient, and less than 1% said that it was more than enough. The variation in responses could reflect variations in the opportunity costs that the compensation was supposed to cover, but strategic bias is likely to have driven some of the negative responses. 36% of respondents said they felt that the distribution of compensation was fair, all of whom were compensation recipients. When asked who is currently receiving compensation, nearly 100% of respondents said the households most dependent on fishing and 59% said the poorest households. However, 73% also chose well-connected people and 20% chose those belonging to fisher associations (S2 Fig)–a result which contradicts the analysis of actual compensation distribution. 99.7% agreed that the most dependent households should be receiving compensation, whereas 0.3% said that every fisher should receive compensation.

The most important fixed effect for the GLMM for perceived fairness was whether, or not, the respondent received compensation; as expected, recipients were significantly more likely to perceive fair compensation distribution (Table 3B). Support for the model was quite weak (S3 Table) and the standard error on this effect size was very large, but the latter was due to convergence issues caused by the absence of any non-recipients who thought the scheme was fair. Fishing dependence also had a weak significant effect; less-dependent households were more likely to say the distribution of compensation was fair (Table 3B). Plotting the BLUPs for each district and each village within district (S3 Fig) showed a significant effect of geography on reported fairness, once fixed effects were taken into account, although the effect of village was much stronger than that of district, which had limited importance.

Given the full compensation coverage in Chandpur, we re-ran these models excluding households in Chandpur (S6 Table). Fixed effects were similar, but the effect of compensation was much lower, and although the effect of fishing dependence remained negative, its relative importance declined from 0.73 to 0.30, while the negative effect of jatka fishing increased in relative importance from 0.25 to 0.50 (S4 Table). The effects of district and village declined to the point that district had almost no effect (S4 Table).

## Discussion

### Compensation distribution

The pattern of compensation distribution in the study area does not strongly reflect the stated social goals of the compensation scheme for hilsa conservation, indicating that it is not targeting its resources as effectively as it could be, or that real objectives may differ from stated objectives. Although the DoF claims to target jatka fishers and those who are fully dependent on fishing, living inside sanctuary areas, evidence only weakly supported the jatka claim and was contrary to the fishing dependence claim. For households outside of Chandpur, living in a sanctuary area had a negative influence on compensation distribution–a result which reflects the fact that Chandpur is both a sanctuary area and has the highest compensation coverage of any district. There was some evidence that larger households with a higher level of food insecurity were more likely to receive compensation, which is coherent with the goals of the VGF programme, but support for these effects was weak. Contrary to expectations, involvement in fisher associations had a negative influence on compensation distribution. Yet, this result should be interpreted with caution since fishing households are known to be largely disorganised in south east Bangladesh [63], overall involvement with fisher associations in the study area was low (6%), and there is evidence to suggest that those which do exist are non-functional [33].

The lack of clarity in the scheme’s targeting criteria makes it difficult to draw conclusions on actual levels of inclusion or exclusion error. Both the proportions of non-fisher and non-hilsa fisher respondents receiving compensation–and the proportions of respondents who were officially listed as recipients but said that they did not receive compensation–were very low. But if the error rates were to be measured through other factors in line with the rationale of the scheme (fishing dependence, income level, or jatka fishing), their lack of influence in statistical models indicates that the error is much higher. For example, although the scheme is aimed at jatka fishers, a similar proportion of recipients and non-recipients said that they target jatka. There is, however, a risk that strategic bias may be driving responses to some questions. It is possible that many more households did not fish, or did not fish hilsa, but declined to volunteer the information due to their understanding of the scheme. Moreover, eliciting honest answers about sensitive behaviours such as illegal resource use is challenging [64] and so it is possible that bias was introduced by concern about the consequences of admitting to the practice of jatka fishing. In this case though, respondents seemed very willing to volunteer the information, and local knowledge suggests that the proportions of jatka fishers and non-jatka fishers in the sample are representative (BH, personal observation).

What this study did reveal is a strong spatial pattern in compensation distribution. Households were significantly more or less likely to receive compensation in some districts than others. Households in Chandpur had the greatest probability of receiving compensation, once other variables were taken into account, a result in line with the fact that all households in Chandpur are eligible for compensation. A range of factors might explain this higher coverage: Chandpur is the district where the scheme was first established and it is considered to be an important landing site for hilsa, so receives a great deal of media attention and relatively good monitoring. It is also thought to be the site of the largest riverine nursery ground for hilsa [26], so it could be argued that, by focusing on this district, ecological objectives are prioritised–assuming the scientific basis for this nursery ground is reliable. It is also possible that households in the coastal districts of Bhola and Patuakhali are less likely to receive compensation because they have less political influence. Moreover, within districts, some villages have more organised and more powerful local councils, and thus more influence in the distribution process. Due to the subjectivity of this process, officials are free to use it for political gain, which has anecdotally been linked to the appearance of non-target recipients on compensation lists, or more than one record per recipient [26]. However, the key role of Chandpur in driving the spatial pattern is demonstrated by the reduced importance of fixed effects (particularly district) when Chandpur households were excluded from model runs.

### Alternative targeting strategies

According to the figures for the study area, it would be possible to compensate all jatka fishers (or those who self-identified as jatka fishers) under current budget constraints. This would result in increased allocation to villages in Patuakhali and Barisal districts, with a decrease to those in Chandpur and Barguna. Yet, although this shift illustrates the current level of mistargeting (Fig 2), the strategy is not a practical one. Firstly, given the sensitivity of the behaviour, the identification of jatka fishers would be challenging. Secondly, the term is a vague one; all artisanal fishers who are dependent on hilsa in inland areas where jatka is abundant would catch jatka, even if it were unintentional. Targeting jatka is also an activity that marginal farmers and labourers often switch to when income is very low, and so many of those targeting jatka are probably seasonal or occasional fishers who do not need compensation to the extent that full-time fishers do [26]. Finally, there is uncertainty around whether focusing on jatka protection is the best strategy from an ecological perspective [65].

Targeting fishing dependence would be a more practical strategy for achieving equitable social impacts through vulnerability reduction [14,63,66]. The implementation of fishing bans disrupts patterns of access to fishing, restricting household flexibility to cope with shocks and thereby contributing to vulnerability [15]. Dependence on hilsa fishing is universally high in and around the sanctuary areas [33], but this study defined three clusters of dependence, and households with different levels of dependence are likely to be differentially affected by loss of fishing access. Under current budget allocation within the study area, around 60% of the most dependent households could be targeted. This would lead to large shifts in the allocation of funds, particularly from households in Chandpur and Barguna to those in Bhola and Patuakhali.

Most of the components used to develop the index of fishing dependence in this study would be possible to verify by those administering the compensation scheme, and some of them–such as household size and boat ownership–are already noted by the DoF as factors in their selection process. One of the most influential variables in the index was the household’s main coping strategy; the most dependent households said that they fish anyway as their main coping strategy during fishing bans. Like jatka fishing, this would not be useful as a characteristic for the identification of compensation recipients since it relates to illegal behaviour and so would not be willingly disclosed to the authorities. To use it would also risk damaging perceptions towards the scheme, through preferentially compensating those who are engaging in illegal behaviour. Nevertheless, a simple scoring system based on the other components of the index could be used to transparently allocate compensation.

There is a risk, in making relief available only to hilsa fishers, of generating a perverse incentive to participate in hilsa fishing. Similar concerns have been raised over a scheme in the Brazilian Amazon that distributes subsidies to artisanal fishers as compensation for a closed fishing season, and it has been demonstrated that this cash compensation has actually contributed to an increase in fisher numbers [1].

For the ecological impact of the scheme to be maximised, there is a need to better understand the role of different areas in the hilsa life cycle. Conservation impact will ultimately depend on whether the regulations and thus compensation are appropriately spatially and temporally targeted, and there is evidence to suggest that the sanctuaries may not be appropriately placed at present. Currently, inland fishers are the focus of regulation and compensation, yet marine fishers appear to have a greater potential ecological impact on hilsa [65,67]. Alongside the social goals of the compensation scheme, explicit ecological goals should be introduced and supported by long-term ecological monitoring.

There are, however, potentially strong trade-offs between ecological and social goals of the scheme. Household impact on hilsa population biomass appears to be driven more by exploitation rate than size selectivity, and there is a lack of correlation between either jatka fishing or high dependence on fishing and catching high volumes of fish [65]. The existence of trade-offs should not prevent interventions from having positive social impacts if they are well designed and implemented, and the trade-offs are made clear from the outset [3,11,12]. But when trade-offs between social and ecological goals are strong, explicit poverty-targeting mechanisms may not be appropriate [68,69]. Instead of attempting to integrate hilsa conservation and poverty alleviation, it might be more appropriate to focus on conservation goals. Given the cost and challenges of effective social assessment in systems like this, where all households are very poor and likely to self-identify as jatka fishers if that means receiving compensation, it could be preferable to compensate all fishers in the areas deemed to have the greatest potential for ecological additionality–currently understood to be those living in and adjacent to sanctuary areas, who are also subject to the greatest restrictions on their livelihoods. An assessment of the relative costs and benefits of this approach versus a more precise social targeting strategy in the hilsa fishery would be worthwhile (Table 4).

**Table 4:** A summary of alternative targeting strategies for the hilsa fisher compensation scheme.

Targeting strategy	Advantages	Disadvantages
Targeting households which pose the greatest ecological threat	• A spatial targeting rule would remove the need for social assessment, which could reduce costs and challenges • High potential ecological additionality, and still has potential to contribute to vulnerability reduction	• Households posing the greatest ecological threat are not necessarily the most vulnerable
Targeting fishing dependence	• Fishing dependence contributes to vulnerability • A scoring system could be used to more precisely and transparently allocate compensation • Greater potential for social acceptability and so potential to incentivize compliance with regulations	• Could generate a perverse incentive to participate in jatka fishing • Trade-offs with ecological goals are strong so low potential for ecological additionality • Effective social assessments are challenging and costly
Targeting jatka fishers	• Should lead to vulnerability reduction	• The term ‘jatka fishers’ is vague and does not necessarily represent the most vulnerable • Vagueness probably limits social acceptability • Jatka fishing is a sensitive behaviour and so identification of these fishers is challenging • Could generate a perverse incentive to participate in jatka fishing • Trade-offs with ecological goals are strong, so low potential for ecological additionality

### Fairness and legitimacy of compensation distribution

The majority of respondents who received compensation (60%) said it was insufficient to compensate for costs incurred by the fishing bans. Although the potential for strategic bias in this response should be taken into account, the result is not surprising. In poor, resource-dependent communities where resource supply is at risk, perceived opportunity costs of behavioural change tend to be very high [70]. Moreover, other studies of the compensation scheme in Bangladesh have found that households tend not to receive their full 40 kg allocation, largely due to shortfalls in distribution costs that local government officials cover by withholding a share of the rice from each allocation [22,26]. Taking the shortfalls into account, these studies suggest that an increase to 50 kg of rice per month should be adequate compensation.

As well as adequately compensating for opportunity costs, a more transparent and precisely targeted scheme may go some way to improving perceptions of legitimacy and fairness. These perceptions are currently poor (64% reporting unfairness) and may be undermining its potential to incentivise compliance with the fishing bans [21,71]. Although the majority of respondents said that they think the most dependent and poorest fishers receive compensation, three-quarters said that the well-connected are also favoured. Procedural legitimacy (deriving from an open and transparent process of decision-making and an explanation of the choices made) is closely linked to perceptions of fairness, and both play an important role in compliance with regulations [16]. Currently, there is none of this legitimacy in the study site.

Aside from the expected association between perceiving unfairness and not receiving compensation, the strongest pattern in perceptions of fairness was between villages. This could be related to the differences in governance that are inferred from the spatial pattern in compensation distribution [17], but the strong effect of district on compensation distribution was much weaker in the model for fairness. No evidence of order bias or other observer effects was found. It should be noted, however, that there may always be perceptions of unfairness when there are winners and losers involved and attitudes are not always strong predictors of actual behaviour [72]. The shift in importance of fishing dependence and jatka fishing after excluding Chandpur households from model runs could be a reflection of the higher proportion of self-identified jatka fishers in other households sampled.

### Compliance with fishing regulations

The limited literature available suggests that compliance with hilsa fishing regulations is poor[22]. Nevertheless, our results do show a small but significant difference in reported compliance between recipients and non-recipients of compensation, which was backed up by respondent perceptions that recipients were more likely to comply. Without baseline data on levels of compliance before the compensation scheme was introduced, we cannot infer causation, but these statistics do indicate that the scheme could be having a small positive impact on compliance [65]. In the absence of any conditionality of compensation on compliance, such as a PES would require, this may be the best that could be hoped for.

## Conclusions

Conservation payments are often specifically targeted according to their goals and objectives. This study reveals a lack of alignment between the stated objectives of the compensation scheme for hilsa fishers in Bangladesh, and the apparent distribution of benefits, highlighting the need for a more focused and transparent targeting strategy. Careful assessment of the potential alternatives (Table 4) should place the distribution more in line with stated objectives, but substantial reform in the way compensation is distributed may not be achievable in the current political climate [26]. These findings contribute to the ongoing debate on whether explicit poverty-targeting can generate good conservation outcomes, highlighting the risk that public conservation payments can be ineffectively targeted, particularly in developing countries, and the issues than can ensue. In circumstances where effective poverty-targeting is politically or financially unfeasible, it may be an inefficient and potentially ineffective approach for achieving additional social or ecological outcomes. Finally, this study demonstrates the value of, and need for, post-hoc evaluations that critically examine whether conservation payments are meeting their stated objectives. If payment schemes are to have social and ecological additionality, they should be properly designed and evaluated. For schemes like this one, which lack baseline data for rigorous impact evaluation, post-hoc evaluations should allow managers to validate their targeting strategy, and ensure that the ‘right’ individuals are receiving benefits.

## Supporting information

S1 Appendix Household survey questionnaire. (PDF)

S2 Appendix Survey design methods. (PDF)

S3 Appendix Development of an index for fishing dependence. (PDF)

S4 Appendix Respondent profile. (PDF)

S1 Fig Best Linear Unbiased Predictors (BLUPs) for the random effects for the probability of receiving compensation. The x axes show the effect of living in a particular district (a) or village (b) in terms of the difference in probability of receiving compensation from the intercept. Error bars show the 95% confidence interval based on the conditional variance for each random effect. Village names are prefixed by district.
(PDF)

S2 Fig Groups of people that respondents perceived to be and thought should be receiving compensation. The total percentage is more than 100 because some respondents (*n* = 799) gave multiple answers.
(PDF)

S3 Fig Best Linear Unbiased Predictors (BLUPs) for the random effects for the probability of reporting fairness of compensation distribution. The x axes show the effect of living in a particular district (a) or village (b) in terms of the difference in probability of reporting fairness from the intercept. Error bars show the 95% confidence interval based on the conditional variance for each random effect. Village names are prefixed by district.
(PDF)

S1 Table Distribution of food grain compensation for hilsa fishers in Bangladesh from 2004 to 2014. Source: DoF 2014.
(PDF)

S2 Table Model selection table for GLMM with probability of receiving compensation. (PDF)

S3 Table Model selection table for GLMM with probability of receiving compensation, excluding Chandpur. (PDF)

S4 Table Results for GLMMs of probability of (a) receiving compensation; and (b) perceiving fair compensation distribution–excluding Chandpur district.
(DOCX)

S5 Table Model selection table for GLMM with probability of perceiving fair distribution of compensation. (PDF)

S6 Table Model selection table for GLMM with probability of perceiving fair distribution of compensation, excluding Chandpur. (PDF)
