# Supplementary material for: Evaluating the ecological and social targeting of a compensation scheme in Bangladesh
Source: PLoS One. 2018 Jun 13;13(6):e0197809. doi: 10.1371/journal.pone.0197809 (PMC5999081; doi:10.1371/journal.pone.0197809)
Supplement: S1 Appendix — (PDF) [file pone.0197809.s001.pdf]

DARWIN Initiative: Economic Incentives to Conserve Hilsa Fish (*Tenualosa Ilisha*) in Bangladesh
International Institute for Environment and Development (IIED),
Bangladesh Centre for Advanced Studies (BCAS) and Bangladesh Agricultural University (BAU)

Questionnaire for Socio-Economic Survey of Affected Fisher HHs under the Project: Payments for Hilsa Conservation in Bangladesh (বাংলাদেশের ইলিশ সংরক্ষন প্রকল্পের ক্ষতিগ্রস্ত মৎস্যজীবী পরিবারের আর্থ-সামাজিক অবস্থা জানার জন্য প্রশ্নপত্র)

Objective of the Socio Economic Study(আর্থ-সামাজিক অবস্থা জরিপ করার উদ্দেশ্য):

- To collect data on socioeconomic characteristics of the "affected" communities(ক্ষতিগ্রস্ত সম্প্রদায়ের আর্থসামাজিক অবস্থা জানার জন্য তথ্য সংগ্রহ করা)
- To assess preferences for compensation packages(ক্ষতিপূরন প্যাকেজের পছন্দ নিরুপন)
- To estimate the opportunity cost of participating in the compensation scheme(ক্ষতিপূরন প্রকল্পে অংশগ্রহনকারীদের ক্ষতির পরিমান জানা)

Name of enumerator (জরিপকারীর নাম): ______________________________ Date (তারিখ): _________________

Code: [number-dd-mm-yy](তারিখের কোড নং) _________________________________

**District**(জেলা)		**Union**(ইউনিয়ন)	
**Upazila**(উপজেলা)		**Village**(গ্রাম)	

Village code: Lalpur-1, Uttar Gobindia-2, Sabujgram-3, Char Laxmi-4, Dakhin Razapur-5, Kalupur-6, Charipara-7, Nizampur-8, Baushia-9, Hizla Gourabdi-10, Baherchar-11, Kutubpur-12, Gosherchar-13, Sajapur-14, Sorab Mondal Para-15, Padma-16, Gunpara-17, Kewrabunia-18,Shoto labongola-19, Charkachopia-20, Rayrabad-21, Mewrapara-22, Chinguria-23, Golbunia-24, Other-25____________

Union Code: Bishnopur-1, Hanerchar-2, Alexander-3, Char Laxmi-4, Dakhin Razapur-5, Ilisha-6, Lalua-7, Mahipur-8, Barojalia-9, Hizla Gourobdi-10, Debogram-11, Doulatdia-12, Muladi sadar-13, Nazirpur-14, Lalmahan-15, Pathorgata sadar-16, Charduani-17, Ailapathagata-18, Burirchar-19, Badarpur-20, Other-21 (Specify)

Upazila Code: Chandpur Sadar-1, Ramgati-2, Bhola sadar-3, Doulatkhan-4, Kalapara-5, Hizla-6, Pathargata-7, Cox's Bazer-8, Muladi-9, Rajbari-10, Barguna Sadar-11, Goalondo-12, Lalmahon-13, Other-14 (Specify)

District Code: Chandpur-1, Laxmipur-2, Bhola-3, Patualkhali-4, Barisal-5, Barguna-6, Cax's Bazer-7, Rajbari-8, Other-8(Specify)

A. Household Characteristics (পরিবারের তথ্য)

1. a. Name of HH Head (খানা প্রধানের নাম) __________________________
   b. Cell No(মোবাইল নাম্বার)----------------------------
   c. Name of Respondent (উত্তর দাতার নাম):_____________________
   d. Relation with HH Head (খানা প্রধানের সাথে উত্তর দাতার সম্পর্ক): __________________________

2. Gender (লিঙ্গ)**0**= Male (পুরুষ)   1= Female (মহিলা)

3. Age (বয়স): _______________ (years)

4. Year of schooling (কত বছর স্কুলে পড়েছেন)-------------------------years

5. Number of people currently living in household (বর্তমানে পরিবারে বাস করে এমন সদস্য সংখ্যা)------------------
Adult male (বয়স্ক পুরুষ) ------- Adult female (বয়স্ক মহিলা)----------Children up to 11 (শিশু ১১ বৎসর পর্যন্ড়) ---------
Adolescents (কিশোর/কিশোরী ১২-১৮ বৎসর পর্যন্ড়) Male (কিশোর)-----------Female (কিশোরী) ______________

6. Other people supported outside of household (পরিবারের বাহিরের কাউকে সহায়তা করেন কিনা)______

7. Number of economically active (earning) household members (পরিবারে আয় করে এমন সদস্য সংখ্যা)_________

8. Main income generating activity (প্রধান আয় বর্ধক কার্যক্রম)_________________

9. Secondary income generatingactivities (দ্বিতীয় আয় বর্ধক কার্যক্রম):______________________

(Professional Code Q8 & Q9: Hilsa/Jatka Fishing(ইলিশ/জাটকা ধরে)-1, Other Fishing (অন্যান্য মাছ ধরে)-2, Day labor (দিন মজুর)-3, Agricultural Activities (কৃষি কাজ)-4, Fish Trading (মাছের ব্যবসা)-5, Service (চাকুরী)-6, Business (ব্যবসা)-7, Handicrafts (হস্ড়শিল্প)-8, Rickshaw/Van puller (রিক্সা/ভ্যান)-9, Farming (গবাদি পশু ও হাঁস মুরগীর খামার)(Livestock)-10, Farming (শাক-সবজীর চাষ)(Vegetables)-11, Tailoring (দর্জির কাজ)-12, Net Repairing /Making (জাল মেরামত/তৈরী)-13, Foreign currency (বৈদেশিক মুদ্রা)-14, Boat man (নৌকার মাঝি)-15, Other (অন্যান্য)(Specify)-16

10. Remittance from family members (পরিবারে র কোন সদস্য বৈদেশিক মুদ্রা আয় করে কিনা)
1= Yes(হ্যাঁ)0= No (না)
If yes, how much per month?_________ Tk

11. Do you have own livestock (নিজস্ব প্রাণিসম্পদ আছে কিনা? 1= Yes(হ্যাঁ)0= No (না)
If yes, then what and how many (যদি হ্যাঁ হয় তাহলে প্রাণিসম্পদের নাম কি এবং কতগুলি? __________
1=Cow-------, 2=Chicken----------, 3= Goat----------, 4= Others (Specify)---------

12. Do you have own land (নিজস্ব জমি আছে কিনা)? 1=Yes(হ্যাঁ)0=No (না)
If yes, then how much (যদি হ্যাঁ হয় তাহলে কত পরিমান)? _________Homestead (বসতবাড়ী)---------Agricultural (কৃষি জমি)-------- Other (অন্যান্য)------- (in local unit of measurement in acre)

13. Have you ever borrowed money from any source (কোন উৎস থেকে ঋন/দাদন নিয়েছেন কিনা)?
1= Yes (হ্যাঁ)0= No (না)
If yes, when was the last time and how much? Last time was in (month and year) ....
Amount: __________ Tk
If No, then why (যদি না হয় তাহলে কেন)?
____
____

14. [if Yes Q13 above, then] who do you usually borrow money from (যদি হ্যাঁ হয়, তাহলে সাধারনত কার কাছ থেকে ঋন/দাদন নেন)?
1=Microfinance institutions (কোন ক্ষুদ্র ঋন প্রদানকারী সংস্থা (e.g. Grameen Bank or other local MFIs) _____Tk
2=Local money lenders (স্থানীয় অর্থলগ্নীকারী) (aratdars and dadondars) ______Tk
3=Relatives and friends (আত্মীয় বা বন্ধুমহল থেকে কোন সুদ ছাড়া) (no interest rate) ______Tk
4=Other (অন্যান্য); please specify __________ and ________ Tk

B. Fishing activities (মাছ ধরা কার্যক্রম)

15. What is the primary purpose of your involvement in fishing (আপনার মাছ ধরা কাজে নিয়োজিত হওয়ার প্রধান উদ্দেশ্য)

1=Subsistence or consumption(খাওয়া/ভোগ)

2=Consumption and selling (খাওয়া এবং বিক্রির জন্য)

3=To sell in the local market, (generate income) (আয়ের জন্য স্থানীয় বাজারে বিক্রি করা)

4=Labour (employed by others) (কর্মসংস্থান / অন্যের কর্মসংস্থান তৈরী)
5=Other? ………………………………….

16. How do you access fishing rights (মাছ ধরার অধিকার কিভাবে পান)?

1=Lease (পত্তন), 2= Share (অংশীদারে), 3= Labour (শ্রমিক), 4= Contract (চুক্তি),

5= License, (সনদ গ্রহণের মাধ্যমে), 6= Free access (উন্মুক্ত)

17. What is your average monthly income from fishing (মাছ ধরা থেকে গড় মাসিক আয় কত)? __________Tk(টাকা)

Month/Season (মাস / সময়)		Monthly income (Tk) (মাসিক আয় টাকা)
1.	March-April(All fishing ban period including jatka) /মার্চ-এপ্রিল (ফাল্গুন-বৈশাখ), জাটকাসহ সকল প্রকার মাছ ধরা নিষেধ	
2.	May-July (normal fishing period) /মে- জুলাই, (বৈশাখ-শ্রাবন) স্বাভাবিক মাছ ধরার সময়	
3.	August-October (Peak fishing period)/আগষ্ট-অক্টোবর, (শ্রাবন-কার্তিক) মাছ ধরার ভরা মৌসুম	
4.	November-January/নভেম্বর-জানুয়ারী (কার্তিক-মাঘ)	
5.	February (Lean fishing period) /ফেব্রুয়ারী, (মাঘ-ফাল্গুন) কম মাছ ধরার সময়	

18. What are the other sources of income of the household(পরিবারে আয়ের অন্য উৎস কি)?

Source of income (আয়ের উৎস)	Yearly income (বাৎসরিক আয়)(Tk)
Total (মোট)	

19. Over the last 5 years has your income from fishing changed (গত পাঁচ বছরে মাছ ধরা থেকে আয়ের কোন পরিবর্তন হয়েছে কিনা)?

1= increased (বৃদ্ধি হয়েছে)2= decreased(কমেছে)3=stayed the same/stable(পরিবর্তন হয় নাই) 4=Not sure (নিশ্চিত না)Why (কেন)?

____

____

20. Are you a member of a fishers association (আপনি কোণ মৎস্যজীবী সমিতির সদস্য কিনা)? 1= Yes (হ্যাঁ) 0= No (না)
If yes, then why (যদি হ্যাঁ হয়, কেন)?

1=Access to information/knowledge (তথ্য আদান প্রদানের জন্য)

2=Access to micro-credit (ক্ষুদ্র ঋনের জন্য)

3=Access to market (বাজারজাত করার জন্য)

4=Access to fishing rights (মাছ ধরার অধিকারের জন্য)

5=other, namely (সাধারণ নিরাপত্তার জন্য) (please specifyনির্দিষ্ট করে লিখতে হবে) _____________

21. 1. Do you own a fishing boat (আপনার নিজস্ব মাছ ধরার নৌকা আছে কিনা)?
1= Yes (হ্যাঁ) 0= No (না)
2. Do you own a fishing net only (আপনার কি মাছ ধরার জন্য শুধু জাল আছে)?
1= Yes (হ্যাঁ) 0= No (না)

22. Do you catch hilsa fish (আপনি কি ইলিশ মাছ ধরেন)?
1= Yes (হ্যাঁ)0= No(না)
If not, then why (যদি না হয়, কেন)?

____

____

23. What types of fishing gear do you use (মাছ ধরার জন্য কি ধরনের সরঞ্জাম ব্যবহার করেন)?

**Sl No** (ক্র:নং)	**Name of Gear** (জালের নাম)	**Target Fish species (Use code)** (লক্ষ্যিত মাছের জাত, কোড ব্যবহার)	**Fishing period (month)** (মাছ ধরার সময়/মাস)	**Location** (মাছ ধরার স্থান)
1	Chandi Jal -2 (large mess) (চান্দি জাল, বড় ফাস)			
2	Chandi Jal-4 (small mess) (চান্দি জাল, ছোট ফাস)			
3	Gulti Jal (গুল্টি জাল)			
4	Current Jal (কারেন্ট জাল)			
5	Behendi net (বেহেন্দি জাল)			
6	Poa Jal (পোয়া জাল)			
7	Chapri Net (চাপরি জাল)			
8	Chewa net (চেওয়া জাল)			
9	Chai (চাই জাল)			
10	Hooks (বড়শি)			
11	Moi Jal (মই জাল)			
12	Masheri Jal (মশাড়ী জাল)			
13	Khot Jal (খোট জাল)			
14	Kachki Jal (কাচকি জাল)			
15	Bata/goara Jal (বাটা জাল)			
16	Pona Jal (পোনা জাল)			
17	Chargherajal (চরঘেড়া জাল)			
18	Cast net (ঝাকি জাল)			
19	Khota Jal (খোটা জাল)			
20	Scoop net (ঠেলা জাল)			
21	Dragnet (চিংড়ি জাল)			
22	Lift net/Khora/Beshal jal (খরা জাল/ভেসাল)			

Sl No (ক্র:নং)	Name of Gear (জালের নাম)	Target Fish species (Use code) (লক্ষ্যিত মাছের জাত, কোড ব্যবহার)	Fishing period (month) (মাছ ধরার সময়/মাস)	Location (মাছ ধরার স্থান)
23	Other (Specify) (অন্যান্য)			

Species code:
Hilsa (ইলিশ)-1, Jatka (জাটকা)-2, Poa (পোয়া)-3, Chewa (চেওয়া)-4, pangas (পাঙ্গাস)-5 boal (বোয়াল)-6 , air(আইর)-7, baghair (বাগাইর)-8, chital (চিতল)-9, foli (ফলি)-10, rita (রীটা)-11, puti (পুটি)-12, tengra (টেংগরা)-13, Golda Chingri (গলদা চিংড়ি)-14, Bagda Chingri (বাগদা চিংড়ি)-15, Pl Bagda (বাগদা পিএল)-16, kakila (কাকিলা)-17, baim (বাইম)-18, bele (বেলে) 19, rui (রুই)-20, Catla (কাতল)-21, mrigel (মৃগেল)-22, kalibaus (কালিবাউস)-23, miror carp (মিরর কার্প)-24, chanda (চান্দা)-25, tepa,potka (টেপা/পটকা)-26, pabda (পাবদা)-27, gutum (গুতম) -28, naftani (নাফতানি)-29, chela (চেলা)-30, mola (মলা)-31, tatkini (টাটকিনি)-32, bata (বাটা)-33, chapila/mamoli (চাপিলা/ম্যামলি)-34, kuchia (কুইচা)-35, bacha (বাচা)-36, Kazali/baspata (কাজলি/বাশপাতা)-37, chaka/gangina (চ্যাকা/গনগনিয়া)-38, dela (ঢেলা)-39, shilong (শিলং)-40, ghaura (ঘাউরা)-41, peali (পিয়ালি)-42, Bajila/batasi (বাজিলা/বাতাশি)-43, tapasi (তাপসি)-44, Kholla(খলন্ডা)-45, Small shrimp (গুড়া চিংড়ি)-46, Kachki (কাচকি)-47, Other অন্যান্য)--48

Month Code: April (বৈশাখ-জৈষ্ঠ্য)-১, May (জৈষ্ঠ্য-আষাঢ়)-২, June (আষাঢ়-শ্রাবন)-৩, July (শ্রাবন-ভাদ্র)-৪, August (ভাদ্র-আশ্বিন)-৫, September (আশ্বিন-কার্তিক)-৬,October (কার্তিক-অগ্রহায়ন)-৭, November (অগ্রহায়ন-পৌষ)-৮, December (পৌষ-মাঘ)-৯, January (মাঘ-ফাল্গুন)-১০, February (ফাল্গুন-চৈত্র)-১১, March (চৈত্র-বৈশাখ)-১২

Location code: Meghna River-1, Tetulia River-2, Andermanik River-3, Agunmukha River-4, Sea-5, Padma River-6, Arial Kha River-7, Bishkhali-8, Payra River-9, Ilisha River-10, Jamuna-11, Other (Specify) -12

____

C. Hilsa trends and status(ইলিশের প্রাপ্তি/ গতি এবং অবস্থা)

24. How does hilsa vary throughout the year/with season (সারা বছর/বিভিন্ন মৌসুমে ইলিশ মাছের কেমন প্রাপ্তি হয়)?[months]

	Peak season (ভরা মৌসুম)	Lean season(কম প্রাপ্তি মৌসুম)	Year to Year (বছর হতে বছর) (1=Increase 2=Decrease 3=Same 4=don't know) (বৃদ্ধি পায়-১ কমে যায়-২ একই রকম-৩ জানিনা-৪)
a) Average catch per fishing trip(kg) (প্রতি ট্রিপে গড়ে মাছ ধরার পরিমান (কেজি)			
b) Fish size (1=S 2=M 3=L) (মাছের সাইজ)			
c) Presence of eggs/fries(ডিমের/ পোনার প্রাপ্তি) (1=Plenty 2=few 3=none)			

25. Over the last 5 years, has your hilsa catch (গত পাঁচ বছরে আপনার ইলিশ ধরার পরিমান)?

    1= Increased (বৃদ্ধি হয়েছে)2=decreased(কমেছে)3=stable(পরিবর্তন হয় নাই)4= Don't know(জানে না)

26. Over the last 5 years hilsa abundance has (গত পাঁচ বছরে ইলিশের মজুদ)?

    1=increased (বৃদ্ধি হয়েছে)2= decreased(কমেছে)3= stable(পরিবর্তন হয় নাই)4= don't know(জানে না)

27. Are you aware of any hilsa management regimes introduced by the government (সরকার ঘোষিত কোন ইলিশ ব্যবস্থাপনা বিষয়ে আপনি জানেন কিনা?

a. Jatka fishing ban period (জাটকা ধরা বন্ধের সময়). 1= Yes(হ্যাঁ)0= No(না)

b. Hilsa sanctuaries (area) (ইলিশ অভয়াশ্রম এলাকা). 1= Yes(হ্যাঁ)0= No(না)

c. Conservation of gravid hilsa(মা -বাবা ইলিশ সংরক্ষন). 1= Yes(হ্যাঁ)0= No(না)

D. The compensation scheme(ক্ষতিপূরন/প্রনোদনা প্রকল্প)

28. [Explain the compensation scheme to respondent (ক্ষতিপূরণ/প্রনোদনা প্রকল্প বিষয়ে উত্তর দাতাদের বুঝিয়ে বলুন)]

a) Are you a recipient of the scheme (আপনি কি এই প্রকল্পের কিছু পেয়েছেন)?

1=Yes (হ্যাঁ)0= No(না)2=Don't know(জানি না)

b). How long have you been participating in the scheme (এই প্রকল্পের অধীনে কত বছর যাবৎ ক্ষতিপূরণ পাচ্ছেন)?

_____________ Years (বছর)

29. In your opinion] do you think the compensation scheme has had positive impact on hilsa stock regeneration (আপনার মতে ক্ষতিপূরন/ প্রনোদনা প্রকল্পের কারনে ইলিশের মজুদে ইতিবাচক প্রভাব পড়েছে কিনা)?

1= Yes (হ্যাঁ) 0= No (না) 2=Don't know (জানে না)

30. [In your opinion] do you think the compensation scheme has had positive impact on hilsa catch levels (ক্ষতিপূরন/ প্রনোদনা প্রকল্পের কারনে ইলিশ মাছ ধরার পরিমানে ইতিবাচক প্রভাব পড়েছে কিনা)?

1= Yes (হ্যাঁ) 0= No (না) 2=Don't know (জানে না)

31. [In your opinion] do you think the compensation scheme has had positive impact on the improvement of fisher livelihoods (ক্ষতিপূরন/ প্রনোদনা প্রকল্পের কারনে মৎস্যজীবীদের জীবনমানের উন্নয়নে ইতিবাচক প্রভাব পড়েছে কিনা)?

1= Yes (হ্যাঁ) 0= No (না) 2=Don't know (জানে না)

32. Over the last 5 years, do you think hilsa stocks have changed as a**direct result of the compensation scheme (ক্ষতিপূরন/ প্রনোদনা প্রকল্পের কারনে বিগত পাঁচ বছরে ইলিশ মাছের মজুদে কোন পরিবর্তন মনে করেন কি)**?

1= Increasing (বৃদ্ধি পেয়েছে) 2=Decreasing (কমেছে) 3= No change (কোন পরিবর্তন হয় নাই)

4= I don't know (জানি না)

If no change, what is the reason (s) **for**(পরিবর্তন না হলে কারন কি)?

-----------------------------.

33. Do you think the distribution of compensation is fair (আপনি কি মনে করেন ক্ষতিপূরনের/প্রনোদনার উপকরন সঠিক ভাবে বিতরন হয়)? 1= Yes (হ্যাঁ) 0= No (না)

a) Why/why not (কেন/কেন না)? ____

34. Which people in the village get compensation(গ্রামের কোন শ্রেনীর মানুষ এই ক্ষতিপূরনের উপকরন পায়)?

1= Poorest (needy) households(গরীব পরিবার)

2= Richest households(ধনী পরিবার)

3= People who fish most(বেশী মাছ ধরে এমন ব্যক্তি)

4= People who are most dependent on fishing for their livelihoods(জীবীকা নির্বাহের জন্য মাছ ধরার উপর অধিক নির্ভরশীল ব্যক্তি)

5= People who are part of fisheries association(মৎস্য সমিতির সঙ্গে সম্পৃক্ত ব্যক্তি)

6= People who are well connected in the village(গ্রামের সাথে ভাল সম্পর্ক আছে এমন ব্যক্তি)

7= I don't know(জানি না)

35. Who do you think should be receiving compensation(আপনার মতে কার এই ক্ষতিপূরন/ প্রনোদনা পাওয়া উচিৎ)? (Choose any)(যেটি পছন্দ)

1=Poorest households (সবচেয়ে বেশী গরীব পরিবার)

2=People who are most dependent on fishing for their livelihoods (মাছ ধরে জীবীকা নির্বাহ করে এমন ব্যক্তি)

3=Every fisherman should receive compensation (সকল মৎস্যজীবীর সুবিধা পাওয়া উচিৎ)

4=I don't know(জানি না)

36. In your opinion, what is the level of compliance (with the ban period and zone) among:(আপনার ধারনামতে জাটকা / ডিমওয়ালা মাছ ধরা বন্ধ আইন কেমন মানে)

**Fisher type**(জেলের ধরন)		**Degree of compliance [tick one for each type of fisher]** (নিয়ম মানার অবস্থা)				
		1=Everybody complies (মানে)	2=Most people comply(অধিকাংশ ব্যক্তি মানে)	3=Some people comply(কিছু ব্যক্তি মানে)	4=Only few people comply(খুবই সামান্য ব্যক্তি মানে)	5=No one complies(একজও মানে না)
**a) Subsistence fishermen**(ভোগকারী মৎস্যজীবী)						
**Commercial fishermen**(জীবীকা নির্বাহকারী মৎস্যজীবী)	**b) Recipient** (গ্রহণকারী)					
	**c) Non-recipient** (অগ্রহণকারী)					
**d) Baddya**(বাইদা)						

37. If not everyone complies in a particular group, then (in your opinion) what needs to be done to enhance compliance within these groups?(নির্দিষ্ট কোন দল যদি আদেশ না মানে তাহলে মানার জন্য কি পদক্ষেপ নেয়া যায়) [Open ended question (উন্মুক্ত প্রশ্ন)]?

38. Do you think the pressure to repay loan is an obstruction to abide with ban period (মাছ ধরা বন্ধকালীন সময় ঋণের টাকা পরিশোধে কোন চাপ থাকে কিনা)?

1= Yes(হ্যাঁ) 0= No(না)

39. Do you think the fishing ban period fits well with the hilsa's breeding season(আপনার মতে ইলিশের ডিম ছাড়ার মৌসুমের সাথে মাছ ধরা বন্ধের সময় সামনঞ্জস্য পূর্ণ কি না)?

1= Yes(হ্যাঁ) 0=No(না) 2= Don't know(জানে না)

E. Coping strategy(পেরে উঠার কৌশল)

40. Is your livelihood affected by the closed/off season and zone (মাছ ধরা/ডিম ছাড়ার সময় মাছ ধরা বন্ধ থাকার ফলে আপনার জীবিকার উপর কোন প্রভাব প্রড়েছে কিনা)?

    1=Yes (হ্যাঁ)0=No(না)

41. How sufficient is the compensation providedপ্রদত্ব ক্ষতিপূরণ/প্রনোদনা কতটুকু যথেষ্ট)?

    1= More than enough (যথেষ্ট) 2=Just enough (মোটামুটি) 3= Not enough (যথেষ্টনয়)

42. If you are not recipient or the compensation is not sufficient, what are the most important strategies you do to cope in times of hardship(আপনি ক্ষতিপূরণের উপকরন পান নাই অথবা যথেষ্ট নয়, তাহলে মাছ ধরা বন্ধ থাকার সময় কস্ট মোকাবেলা করেন কিভাবে)? Please rank the5 most frequently used coping strategies [9](দয়া করে মাছ ধরা বন্ধকালীন সময়ে সংসার পরিচালনার কৌশল বিন্যাস করুন, ১-৯ পর্যন্ড়)Rank at least up to 5

Coping strategies (পেরে উঠার কৌশল)	Ranking(বিন্যাস)
1=Use my savings(জমানো টাকা ব্যবহার করে)	
2=Borrow from relatives/friends/others(আত্নীয়/বন্ধু/অন্যান্য কারও নিকট থেকে ধার নিয়ে)	
3=Loan with interest (সুদে ঋণ)	
4=Shopping credit (দোকান বাকী)	
5=Do some other job (e.g. labour or rickshaw/petty business)(অন্য কোন কাজ করে যেমন-দিন মজুর, রিক্সা চালানো)	
6=Consume less preferred and less expensive food(কম চাহিদার ও কম দামী খাবার খান)	
7=Limit portion size at meal times(কম খান)	
8=Sell some assets (livestock if any)(কিছু সম্পদ বিক্রয় করে যেমন-গবাদিপশু, হাঁস-মুরগী ইত্যাদি)	
9=Other (specify)(অন্যান্য)	

F. Opportunity Cost Questions(সুযোগ হারানো সম্পর্কিত প্রশ্ন)

43. Has not being allowed to catch hilsa in particular zones during the ban period changed your fishing behaviour (মাছ ধরা বন্ধ ঘোষিত এলাকায় মাছ ধরতে না দেওয়ায় আপনার মাছ ধরার ক্ষেত্রে কোন পরিবর্তন হয়েছে কি)?

    1) Yes (হ্যাঁ) 0= No(না)

    If, yes, then

    1= changed location (স্থান পরিবর্তন)

    2= changed location and gears (স্থান ও জালের পরিবর্তন)

    3= changed target species (লক্ষ্য মাছের পরিবর্তন)

    4= don't fish during the ban period (বন্ধকালীন সময় কোন মাছ ধরেন না)

    5= I go fishing anyway(যে কোন মূল্যে মাছ ধরতে যাই)

    6=others/specify (অন্যান্য উলেণ্ডখ্য করুন) ____________________

44. If so, has this had an effect on your household income(এই পরিবর্তনের কারনে আপনার পরিবারের আয়ের উপর কোন প্রভাব পড়েছে কিনা)?

    1=Increased(বৃদ্ধি হয়) 2=decreased(কমে যায়) 3=stayed the same(কোন পরিবর্তন হয় না)

    4=not sure (নিশ্চিত নয়)

45. How much in household income do you lose directly due to the ban period and zone(মাছ ধরা বন্ধ ঘোষনার জন্য পরিবারের সরাসরি কত টাকা ক্ষতি হয়)? This includes wages (if employed by other fishermen), or loss in earnings from selling fish(শ্রমিকের মজুরী অন্তর্ভূক্ত করতে হবে, যদি সে শ্রমিক জেলে হয় অথবা মাছ বিক্রি করে).

______________________ Taka per closure period [Jatka](জাটকা ধরা বন্ধ সময়ে টাকার পরিমান)

______________________ Taka per closure period [brood](মা মাছ ধরা বন্ধ সময়ে টাকার পরিমান)

46. How much in household income do you get**<u>from other activities that you do during the ban period, which you wouldn't have done if you had been fishing</u>**(not things that your household would have done anyway)(মাছ ধরা বন্ধকালীন সময়ে অন্য কাজ যা মাছ ধরা সময়ে করে না, তা করে কত টাকা আয় করেন)?

    ______________________ Taka per closure period(প্রতি বন্ধকালীন সময়ের টাকার পরিমান)

47. If you are affected by the current ban period (or if you were to be affected in the future) how much is your minimum willingness to accept compensation in Taka?(আপনার বর্তমান মাছ ধরা বন্ধের কারনে যে ক্ষতি হয় তা পোষানোর জন্য সর্বনিম্ন কত টাকা ক্ষতিপূরণ প্রয়োজন)

    _________________Taka per year(টাকা প্রতি বছর)

Eliciting preferences

(Open-ended question followed by choice experiment)

<u>STANDARD TEXT FOR THE INTERVIEWERS TO BE READ OUT LOUD OR MEMORIZED WORD BY WORD, SO EVERY RESPONDENT IS TOLD EXACTLY THE SAME THING!</u>

As you may know, in response to the decline in hilsa catch levels, the Department of Fisheries of the Government of Bangladesh has introduced hilsa sanctuaries and a no-take season. A limited ban on fishing of jatka, and restrictions on the catching of brood hilsa during breeding season were adopted in 2003 and 2004. Currently this includes (1) a ban of jatka catching and selling/buying from Nov until June; (2) up to 3 months of complete ban of fishing in the sanctuaries; and (3) a ban on catching brood hilsa 5 days before and after full moon in the month of *Ashvin*. To compensate for the loss in earnings due to the ban period, the government started providing affected fisher communities with 30 kilograms of rice per household per month for 4 months and supporting alternative income-generating activities.

I would now like you to answer a series of questions related to existing and possible future bans on fishing <u>inside the sanctuaries</u> and related compensation, in kind and in cash. I will show you 6 cards which specify the number of months during which you cannot fish jatkaand the number of days during which you cannot catch brood hilsa inside the sanctuaries. On every card you may see a different number of months and days, because we want you to carefully think about the consequences of different bans on your household’s livelihood. The number of months can vary between 4, 8 and 12 months where 12 months means a complete ban on jatka fishing for an entire year, and the number of days can vary between 5, 11 or 30 days where 30 days means that you cannot fish brood hilsafor the entire month of Ashvin.

In return you are offered compensation for your lost earnings: in kind through rice – 30 kg per household per month for 4 months - or through supporting you to earn money in alternative ways:

you will either get a rickshaw to start your own business or a sowing machine to earn money from making clothes. You can choose which one of the two you prefer.

You will also be offered money. This can vary between 6, 12, 18 or 24 thousand Taka in total. You can also choose when you wish to receive the money compensation: all in one time (so 6, 12, 18 or 24 thousand Taka in one time), or divided per month or 6 months. For example, if you would be offered 12 thousand taka in total, instead of getting this money amount all at once, you can also receive 1000 thousand Taka on a monthly basis for 12 months or receive 6 thousand Taka in 6 months time and another6 thousand Taka after 12 months.

All we are interested to know from you is which alternative you prefer. I will show you 6 cards and every card contains every time 2 alternatives from which you have to choose. The alternatives differ from each other. Every time I show you a card, I want you to tell me which alternative you prefer, independently from the alternatives and choices you made before. So you make a new choice with every card that I show you. If you do not like any of the 2 alternatives, you can also choose “none of the 2”. In that case you will not participate in the program and also not receive any compensation, not in kind or in cash.

It is very important that you clearly understand each alternative (compensation package) before you state your most preferred alternative. The results from this survey will be used to inform decision-makers to see if the current compensation scheme can be improved. So, we want you to answer as truthfully as possible and as if you would actually receive the outcome of the alternative of your choice. I will first show you an example card and explain to you what the alternatives mean. I will then ask you to choose your most preferred alternative. After this example card, I will show you 6 other cards, and every time all you have to do is carefully look at the alternatives and choose the one you prefer most.

[Present 6 slides one by one. And repeat the first slide. This means 7 slides in total.

Each respondent will be shown 6 choice cards + first card again at end as card 7

Each choice cards has 2 alternatives plus 1 opt out (none of the two)

প্রনোদনা নির্বাচনের পূর্বে ব্যাখ্যা করার বিষয় বস্তু নিম্নে লেখা হলো:

ইলিশের উৎপাদন উলেণ্ঢখযোগ্য হারে হ্রাস পাওয়ার কারণে বাংলাদেশ সরকারের মৎস্য অধিদপ্তর ইলিশ মাছের অভয়াশ্রম প্রতিষ্ঠা এবং নির্দ্দিষ্ট সময়ে জাটকা না ধরার কার্যক্রম চালু করেছে। জাটকা ইলিশ এবং ইলিশ মাছের প্রজনন মৌসুমে মা ইলিশ ধরার উপর ২০০৩-৪ সাল থেকে সরকারি নিষেধাজ্ঞা আরোপ করা হয়। বর্তমানে অর্ন্তভূক্ত আছে (১) নভেম্বর-জুন মাস পর্যন্ড় ঝাটকা ধরা, ক্রয়-বিক্রয় বন্ধ (২) অভয়াশ্রম এলাকায় সরকারি ভাবে তিন মাস সকল প্রকার মাছ ধরার উপর নিষেধাজ্ঞা আরোপ করা হয়েছে। (৩) ইলিশের প্রজনন মৌসুমে অর্থাৎ আশ্বিন মাসের পূর্ণিমার ৫ দিন আগে ও পরে ডিমওয়ালা ইলিশ ধরা সরকারি ভাবে নিষেধ। সরকারি নিষেধাজ্ঞার সময় জেলেদের উপার্জনে লোকসান হওয়ায় ক্ষতিপূরণ দিতে সরকার ক্ষতিগ্রস্থ জেলে সম্প্রদায়কে প্রতি মাসে ৩০ কেজি করে মোট চার মাস চাল দিচ্ছেন এবং তাদের বিকল্প কাজের মাধ্যমে উপার্জনক্ষম করার জন্য সহায়তা করছে।

আমি এখন আপনাকে বর্তমান প্রচলিত এবং ভবিষ্যতে ইলিশ ও জাটকা ধরার উপর সম্ভাব্য নিষেধাজ্ঞা এবং এর প্রেক্ষিতে ক্ষতিপূরন হিসেবে খাদ্য ও নগদ টাকা প্রদান সম্পর্কে কিছু প্রশ্ন করব।

আমি আপনাকে ৬টি কার্ড দেখাবো, যাতে কয় মাস জাটকা মাছ ধরতে পারেন না এবং অভয়াশ্রমে কয় দিন মা মাছ ধরতে পারেন না তা রিপিবদ্ধ করা আছে। প্রত্যেক কার্ডের মাস ও দিনের সংখ্যা এক রকম নয়, কারন মাছ ধরার উপর বিভিন্ন সময়কালের নিষেধাজ্ঞা আপনার পরিবারের উপর কি প্রভার ফেলে তা যাতে আপনি ভালভাবে চিন্ড়া করতে পারেন। জাটকা মাছ ধরার মাসের নিষেধাজ্ঞা ৪ মাস, ৮ মাস এবং ১২ মাসের (সারা বছর) মধ্যে পার্থক্য হতে পারে এবং মা ইলিশ ধরার নিষেধাজ্ঞা ৫ দিন, ১১ দিন এবং ৩০ দিন (পুরো আশ্বিন মাস) হতে পারে।

মাছ ধরতে না পারার কারনে যে ক্ষতি হয় তার ক্ষতি পূরন হিসেবে আপনাকে (১) চাউল ৩০ কেজি /পরিবার/ প্রতি মাসে মোট ৪ মাস অথবা (২) বিকল্প আয়ের জন্য রিক্সা দিয়ে নিজের ব্যবসা শুর“ করা অথবা সেলাই মেশিন দিয়ে কাপড় তৈরী করে টাকা আয়ের ব্যবস্থা করা হয়। আপনি এই ২টি ব্যবস্থা থেকে যে কোন ১টি ব্যবস্থা পছন্দ করতে পারেন।

অথবা (৩) আপনকে নগদ টাকাও দেয়া হতে পারে। এই টাকার পরিমান হতে পারে ৬০০০ টাকা, ১২০০০ টাকা, ১৮০০০ টাকা এবং ২৪০০০ টাকা। এখন এই টাকা আপনি কিভাবে পেতে চান? একই সময়ে চান, নাকি ৬ মাসে চান নাকি ১২ মাস দিয়ে ভাগ দিয়ে মাসে চান। যেমন- ১২ মাসে দিয়ে ভাগ করলে প্রতি মাসে ১০০০ টাকা অথবা ৬ মাসের জন্য ৬০০০ টাকা হলে মাসে ১০০০ টাকা।

আমরা এখন জানতে চাই যে, বিকল্প কাজ হিসেবে আপনি কি পছন্দ করছেন। আমি আপনাকে ৬টি কার্ড দেখাবো এবং প্রত্যেক কার্ড এ ২ রকম ক্ষতিপূরন আছে, যাহা হতে আপনি ১টি পছন্দ করতে পারেন। প্রত্যেক কার্ডে বিভিন্ন প্রকার ক্ষতি পূরন ব্যবস্থা থাকবে। প্রত্যেক সময় আমি আপনাকে ১টি কার্ড দেখাবো এবং আমি চাই আপনি আপনার বিকল্প ক্ষতিপূরন/প্রনোদনা পছন্দ স্বাধীন ভাবে বলতে পারেন। আমি যখন আপনাকে পূণরায় ১ম/নতুন কাডর্টি দেখাবো তখনও আপনি যেন আপনার পছন্দ বলতে পারবেন। আপনি যদি ২টির একটিও না চান তাহলে আমরা বুঝব অআপনি কোন ক্ষতিপূরন/প্রনোদনা চান না।

আপনি পছন্দ করার নির্বাচন কতরার আগেই ভাল করে বুঝবেন। আমি আপনাকে উদাহরন স্বরূপ একটি কার্ড দেখাবো এবং বিকল্পগুলি কি তা বুঝাবো। তারপর আমি ৬টি কার্ড দেখাবো এবং প্রত্যেক বারে আপনি বুঝে এ থেকে যে কোন একটি অধিকতর পছন্দের বিকল্প/প্রনোদনা বেছে নিবেন।

প্রত্যেক উত্তরদাতাকে ছয়টি কার্ড দেখাতে হবে এবং পূনরায় প্রথমটি দেখাতে হবে।
প্রত্যেক পছন্দের কার্ডে দুইটি বিকল্প থাকবে এবং এ থেকে উত্তর দাতা একটি বেছে নেবে।

INTERVIEWER INSTRUCTIONS(প্রশ্নকারীর জন্য নির্দেশনা)**:**

1) FIRST SPECIFY CHOICE SET NUMBER (1-10) (IMPORTANT!!!) (প্রথমে পছন্দের নম্বরটি লিখতে হবে)**: ....**

2) CROSS/CIRCLE THE ALTERNATIVE CHOSEN BY THE RESPONDENT ON EACH CARD IN THE TABLE BELOW(উত্তর দাতার প্রত্যেক পছন্দের বিকল্পটি নিম্নের কলামে লিখতে হবে)

**Card number (1, 2, 3, …, 10)** ________	**Alternative 1**	**Alternative 2**	**None of the two**

Card 1	1	2	3
Card 2	1	2	3
Card 3	1	2	3
Card 4	1	2	3
Card 5	1	2	3
Card 6	1	2	3
Card 7 = Card 1	1	2	3

48. What was **the most important** feature on each card to choose between the presented alternatives? (প্রত্যেকটি চিত্রের উপস্থাপিত বিকল্প প্রোনোদনা নির্বাচনে কোন বিষয়টি অধিকতর গুরুত্বপূর্ণ ছিল)

1=ban jatka fishing (জাটকা ধরা বন্ধ)

2=ban hilsa fishing (ইলিশ ধরা বন্ধ)

3=in-kind compensation (উপকরন প্রোনোদনা)

4=cash compensation (নগদ টাকা)

5=payment frequency (বিতরণের সময়কাল)

49. What was **the least important** feature on each card to choose between the presented alternatives? (প্রত্যেকটি চিত্রের উপস্থাপিত বিকল্প প্রোনোদনা নির্বাচনে কোন বিষয়টি সবচেয়ে কম গুরুত্বপূর্ণ ছিল)

1=ban jatka fishing (জাটকা ধরা বন্ধ)

2=ban hilsa fishing (ইলিশ ধরা বন্ধ)

3=in-kind compensation (উপকরন প্রোনোদনা)

4=cash compensation (নগদ টাকা)

5=payment frequency (বিতরণের সময়কাল)

50. Did you pay equal attention to all choice cards? (সকল পছন্দ কার্ডে সমান মনোযোগ দিয়েছে কিনা)

0=no 1=yes

51. If not, can you explain why not? (যদি না হয়, ব্যাখ্যা দিতে হবে)

____

____

52. Do you believe that you will actually receive the compensation as presented in the choice alternatives? (আপনি কি বিশ্বাস করেন যে, প্রকৃত পক্ষে পছন্দ কার্ডে উপস্থাপিত বিকল্প প্রনোদনাআপনি পাবেন ?)

0= no 1= yes

INTERVIEWER INSTRUCTION: ONLY ASK THIS QUESTION IN THE VERSION WITH PICTOGRAMS (**প্রশ্নকারীর জন্য নির্দেশনা: শুধু মাত্র ছবিযুক্ত প্রশ্নপত্রে এই প্রশ্ন করতে হবে**)

53. What role did the visualizations of the different features or characteristics play in your decision-making? (বিভিন্নপ্রকার ছবি ক্ষতিপূরন পছন্দে আপনার সিদ্ধান্ড় গ্রহণে কেমন ভূমিকা রেখেছে)

0= No role at all (মোটেও ভূমিকা রাখে নাই)

1= somewhat of a role (অল্প কিছু ভূমিকা রেখেছে)

2= A big role (অনেক বেশী ভূমিকা রেখেছে)

54. If the visualizations played somewhat or a big role, can you explain how? (ছবিগুলো যদি কম-বেশী কোন ভূমিকা রেখে থাকে তাহলে কেন কম-বেশী ভূমিকা রেখেছে তা ব্যাখ্যা করতে পারবেন ?)

1= They drew all of my attention or they drew my attention away from the other features or characteristics, especially the pictogram for…………………. (১=এই ছবিগুলি আমায় সম্পূর্ন মনোযোগ আকর্ষণ করেছে অথবা এই ছবিগুলি অন্য ছবিগুলো থেকে আমার মনোযোগ দূরে সরে নিয়ে গেছে , যে ছবিটি দূরে সরে নেয়, সে ছবিটি ফাকা স্থানে লিখতে হবে)

2= Other(অন্য কি ভূমিকা রেখেছে), namely (নামগুলি): ……………………………………………………………………………
