# Supplementary material for: Evaluating the ecological and social targeting of a compensation scheme in Bangladesh
Source: PLoS One. 2018 Jun 13;13(6):e0197809. doi: 10.1371/journal.pone.0197809 (PMC5999081; doi:10.1371/journal.pone.0197809)
Supplement: S2 Appendix — (PDF) [file pone.0197809.s002.pdf]

Within the selected districts, sub-districts (upazila) with a high concentration of fishers were identified through consultations with District Fisheries Officers (DFOs) and Upazila Fisheries Officers (UFOs). Due to difficulties achieving a random and conventional sample design, we aimed instead to interview a large enough proportion of the total fishers in each upazila, so that any problems associated with small and non-random sampling were not likely to influence results. For each selected upazila, villages with a high concentration of hilsa fishers and located near the river were then selected, based on the list of fishers. Each upazila sample was then allocated proportionately to the selected villages, according to the total number of fishers in the village. All households in the selected village were serially numbered and every n^{th} household was selected for interview, where n is the total number of fishers in the village divided by the total upazila sample size. For example, in Chandpur District, two villages were selected: Lalpur and Gobindia villages. The total number of hilsa fishers in Lalpur and Gobindia were 143 and 737 respectively. The total number of hilsa fishers in Chandpur (880) was divided by 150 (target sample size for Chandpur district). Therefore, an interval of six was used to select interviewees from the list of fishers.

Within a household, enumerators interviewed the fisher, if available. If not, they chose a male household member over a female member, since they tend to be more involved in fishing activities. If the enumerators could not find anyone to speak to in the selected household, or if the ratio of recipients to non-recipients in compensation areas was becoming unbalanced, they selected the preceding or following fisher on the list.

Data were collected on opportunity costs by asking respondents to estimate a) how much household income they lose as a direct result of to the fishing ban (and any extra income they get during that period from activities they would not have done outside of the fishing ban); and b) their monetary willingness to accept compensation (see questions 45-47 of the household survey questionnaire, Appendix S1). These data could have been incorporated into a trade-off analysis with fishing dependence and potential ecological impact. However, they were discarded when preliminary analysis showed little convergence between the two proxies of opportunity cost, and high heterogeneity; these kinds of questions are very leading and the proxies they generate are notoriously inaccurate.

Table A. Details of survey sites with the total numbers of fishing households in each, the numbers of compensation recipients and non-recipients, and sample sizes.

District	Upazila	Village	Fishing households	Recipients	Non- recipients	Sample size		
						Recipients	Non-recipients	Total
Chandpur	Chandpur Sadar	Lalpur	143	143	0	25	0	25
		Uttar Gabindia	737	737	0	125	0	125
		Sub-total	880	880	0	150	0	150
Laxmipur	Ramgati	Sabujgram	1186	782	404	25	48	73
		Char Laxmi	1480	1190	290	25	52	77
		Sub-total	2666	1972	694	50	100	150
Bhola	Bhola Sadar	Dakhin Razapur	790	425	365	10	30	40
		Kalupur	659	24	635	20	15	35
	Lalmohan	Char Kachopia	865	459	406	16	36	52
		Rayrabad	400	268	132	5	18	23
		Sub-total	2714	1176	1538	51	99	150
Patuakhali	Kalapara	Charipara	320	160	160	5	13	18
		Nizampur	1475	737	738	25	50	75
		Golbunia	302	150	152	5	10	15
		Mewrapara	598	298	300	12	18	30
		Chinguria	280	140	140	5	7	12
		Sub-total	2975	1485	1490	52	98	150
Barisal	Hizla	Baushia	899	727	172	37	20	57
		Hizla Gourbdi	325	263	62	13	5	18
	Muladi	Kutubpur	55	20	35	5	15	20
		Goswherchar	78	40	38	28	2	30
		Sub-total	1357	1050	307	83	42	125
Barguna	Patharghata	Padma	444	186	258	20	15	35
		Gunpara	515	216	299	29	11	40
		Sub-total	959	402	557	49	26	75
		Grand total	11551	6965	4586	435	365	800

Village statistics were obtained from [1].

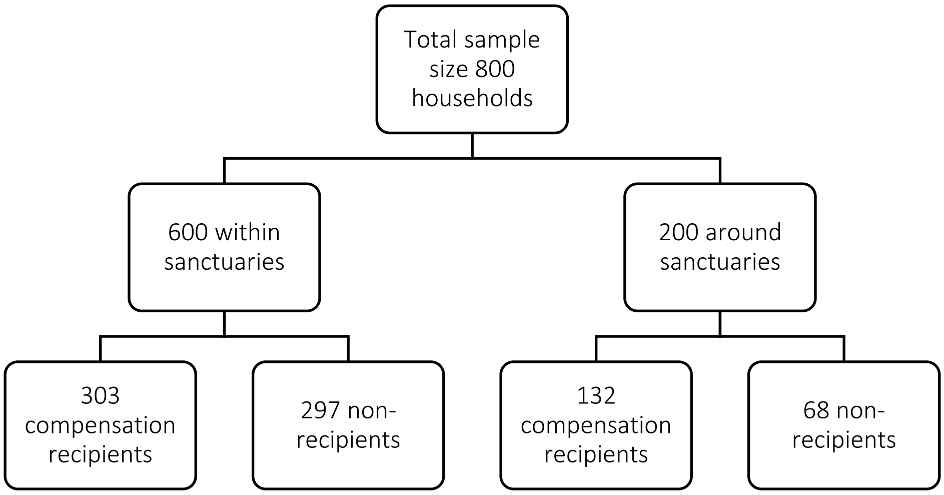

Fig A. Household sampling design.

References

1. Department of Fisheries. Hilsa fishing village statistics. 2014.
