# Supplementary material for: Evaluating the ecological and social targeting of a compensation scheme in Bangladesh
Source: PLoS One. 2018 Jun 13;13(6):e0197809. doi: 10.1371/journal.pone.0197809 (PMC5999081; doi:10.1371/journal.pone.0197809)
Supplement: S3 Appendix — (PDF) [file pone.0197809.s003.pdf]

Dependence is a multidimensional concept and therefore difficult to measure. Dependence on a resource or occupation is not necessarily reflected by level of use because there may be equally good alternatives [1]. Instead, dependence implies that there is no equivalent substitute for the resource or occupation without a loss in wellbeing. In order to explore the relationship between fishing dependence and compensation allocation, we constructed an index for fishing dependence. The development of the index was data-driven, but we used only variables which a priori were known to play a meaningful role in fishing dependence, based on the literature (Table A). We presumed a household to be dependent on fishing when it was the primary livelihood in terms of both income and number of livelihoods. A household with high livelihood diversity may still derive the majority of their income from fishing, and a household with low livelihood diversity may derive similar levels of income from each livelihood [2]. Income dependence was measured using the proportion of household income coming from fishing, while livelihood diversity was measured using the number of livelihoods together with ownership of livestock and agricultural land.

We also expected boat owners to be more dependent on fishing; if capital is invested in fishing, this is expected to reduce adaptive capacity to move away from fishing and therefore increase dependence [3]. Gear diversity is expected to have a similar effect: although using multiple gears reduces the level of specialisation within fishing [1,4], it is still assumed to increase dependence on fishing in general through capital investment. Finally, we expected households that said they fish anyway as their main coping strategy during fishing bans to be more dependent on fishing than those which did not.

Principal component methods are commonly employed to aggregate multiple variables in the development of indices for multidimensional concepts, particularly wealth [5–10]. Application of Principal Component Analysis (PCA) has allowed the quantification of relative household and community vulnerability to shocks such as climate change, with implications for conservation and development policy interventions [11–13]. Moreover, the validity of PCA in the measurement of vulnerability, resilience, and fishing dependence within fishing communities has recently been demonstrated [1,14,15].

However, PCA is designed for use with continuous, normally distributed data [16] and as such has been criticised for use in the construction of indices using discrete data [17,18]. Instead we used factor analysis for mixed data (FAMD), a principal component method that can balance the influence of continuous and categorical variables [19,20], in the R package FactoMineR [21,22]. Following the methods of [8], we carried out descriptive analyses to inform final variable selection, only selecting variables that were significantly correlated ($p < 0.05$) with the majority of the others.

We used the first dimension as a multivariate indicator of dependence, as is the convention in the construction of socioeconomic indices [8,18]. The first dimension explained 30.2% of total variation and can easily be interpreted as a measure of fishing dependence, whereas the second dimension is more difficult to interpret (Table B). It should be noted that it does not represent a complete measure of fishing dependence; with more data, other studies have been able to develop indices that explain over 70% of variation [1]. However, it does exceed the 30% threshold necessary to avoid misclassification [23]. The index ranged from -3.44 to 2.63, so we rescaled it (-1 to +1) for more intuitive interpretation.

Table A. Summary of variables used to develop an index of fishing dependence through FAMD.

Variable	Type	Description	Expected influence	Explanation
Income dependence	Percentage	The proportion of total household income which comes from fishing	+	The greater the share of fishing income in a household's total income, the more dependent the household is on fishing [2,24–26]
Other livelihoods	Binary	Households may have other livelihoods (1) or only fishing (0)	-	The more livelihood options a household has, the more able it will be to adapt to or cope with loss of fishing access [2,4,25,27]
Agricultural land	Binary	Households may have agricultural land (1) or not (0)	-	Households with land may be more able to cope with loss of fishing access [28]
Livestock	Binary	Households may have livestock (1) or no livestock (0)	-	Households with livestock may be more able to cope with loss of fishing access [28]
Boat	Binary	Households may own a boat (1) or not (0)	+	It is often the fishers who have invested in the fishery who are least able to adapt to loss of access as they have sunk their capital into fishing [3]
Gear diversity	Binary	Households may use one gear type (0) or multiple gear types (1)	+	Less highly specialised fishers, with multiple gears, are likely to be more resilient within their fishing livelihood but less able to adapt to complete loss of fishing access [1,4]
Illegal fishing	Binary	Fish illegally as main coping strategy during fishery closure (1) or other coping strategy (0)	+	Fishers who admit fishing illegally over other coping strategies may have few alternatives [29,30]

To check for internal coherence of the index we performed an agglomerative hierarchical cluster analysis on the first dimension of the FAMD, with Euclidian distance and Ward's criterion in FactoMineR [22]. This is a preferable approach to defining arbitrary cut-off points [9]. Households were thus differentiated into broad groups by their level of dependence, which also allowed for more intuitive interpretation. Hierarchical clustering defined a three-cluster solution (Fig A). Fishing dependence differed significantly between these clusters (ANOVA; $\eta^2 = 0.86$; $p < 0.001$) and the difference between mean dependence was highest between the medium and high clusters (Table C). A comparison of means for each constituent variable of the dependence index between clusters demonstrates internal coherence (i.e., each variable is linked to the cluster it is in; Table D). All the constituent categorical variables were significantly linked to cluster (chi squared tests; $p < 0.001$), apart from livestock, which followed no clear trend and did not appear to contribute much to the index. Percentage income from fishing was also significantly linked to cluster (ANOVA; $\eta^2 = 0.52$; $p < 0.001$). There was a weak but significant negative correlation between dependence and average monthly household income (Pearson's $r = -0.17$, $p < 0.001$), which provides some validation for the use of the index as a measure of fishing dependence; low income can increase the level of dependence on a given livelihood [1,24,31].

We repeated the modelling of compensation allocation using dependence cluster as an ordinal explanatory variable instead of the index, to check for consistency, but results were similar so only the analysis using the index is reported in the paper.

Table B. Variables used in FAMD to calculate a fishing dependence score for all surveyed households.

Variable	Units	Dim 1	Dim 2	Dim 3
Illegal fishing	1 = yes, 0 = no	1.3	-0.1	0.1
Boat ownership	1 = yes, 0 = no	0.3	0.4	-0.1
Gear diversity	1 = > 1 type, 0 = 1 type	0.5	0.2	0.7
Agricultural land	1 = yes, 0 = no	-0.6	2.2	1.0
Livestock	1 = yes, 0 = no	-0.0	0.6	-0.4
Other livelihoods	1 = yes, 0 = no	-0.6	0.1	0.1
Fishing income	BDT	0.8	-0.0	-0.1
Eigenvalue		2.1	1.3	1.0
Cumulative variance explained (%)		30.2	48.0	61.6

Principal component coefficients are presented for the first three dimensions.

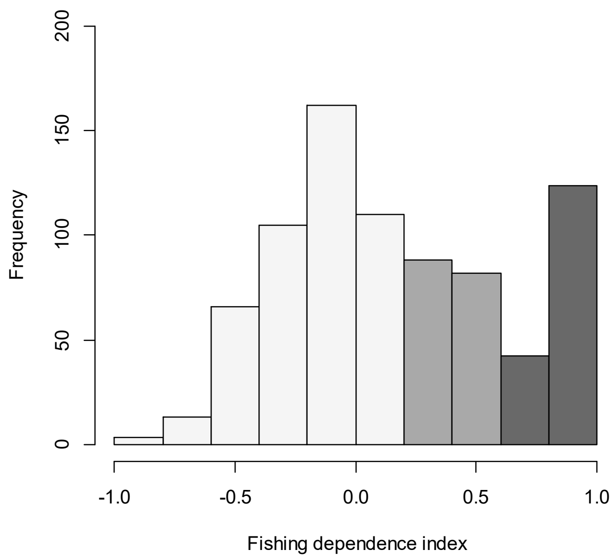

Fig A. Histogram showing the distribution of fishing dependence across households. 1 is most dependent and -1 is least dependent. The different shading differentiates groups of dependence identified through cluster analysis.

Table C. Proportion of households in each cluster of fishing dependence and mean fishing dependence scores for the entire sample.

Cluster	Low dependence	Medium dependence	High dependence
Proportion of households (%)	46.4	32.6	21.0
Mean dependence score	-0.2	0.3	0.9

Table D. Summary statistics and description of clusters of fishing dependence by constituent variables.

	Units	Mean (SD)	Means by cluster<br>Low dependence	Medium dependence	High dependence
Fishing income	BDT	82.3 (16.8)	71.7	87.6	99.7
Agricultural land	1= yes, 0 = no	0.05 (0.2)	0.1	0.1	0.1
Other livelihoods	1= yes, 0 = no	0.8 (0.4)	1.0	1.0	0.02
Boat ownership	1= yes, 0 = no	0.7 (0.5)	0.5	0.8	0.8
Multiple gear types	1 = > 1, 0 = 1	0.5 (0.5)	0.3	0.7	0.7
Illegal fishing	1= yes, 0=no	0.4 (0.5)	0.02	0.7	0.9
Livestock	1= yes, 0=no	0.6 (0.5)	0.6	0.6	0.6

The standard deviation (SD) is presented in brackets.

References

1. Marshall NA, Fenton DM, Marshall PA, Sutton SG. How Resource Dependency Can Influence Social Resilience within a Primary Resource Industry. Rural Sociol. 2007;72: 359–390. doi:10.1526/003601107781799254

2. Hill N. Livelihood diversification for conservation: Interactions between seaweed farming and fishing in Danajon Bank, central Philippines. Ph.D. Thesis. Imperial College London. 2011.

3. Blythe JL, Murray G, Flaherty M. Strengthening threatened communities through adaptation: insights from coastal Mozambique. Ecol Soc. 2014;19.

4. Cinner JE, McClanahan TR, Graham N a. J, Daw TM, Maina J, Stead SM, et al. Vulnerability of coastal communities to key impacts of climate change on coral reef fisheries. Glob Environ Chang. 2012;22: 12–20.

5. Filmer D, Pritchett LH. Estimating wealth effects without expenditure data — or tears: an application to educational enrolments in states of India. Demography. 2001;38: 115–132.

6. Mckenzie DJ. Measuring Inequality with Asset Indicators. Pop Econ. 2004;18:229-260.

7. Zeller M, Sharma M, Henry C, Lapenu C. An operational method for assessing the poverty outreach performance of development policies and projects: Results of case studies in Africa, Asia, and Latin America. World Dev. 2006;34: 446–464. doi:10.1016/j.worlddev.2005.07.020

8. Vyas S, Kumaranayake L. Constructing socio-economic status indices: how to use principal components analysis. Health Policy Plan. 2006;21: 459–68. doi:10.1093/heapol/czl029

9. Lalloué B, Monnez J-M, Padilla C, Kihal W, Le Meur N, Zmirou-Navier D, et al. A statistical procedure to create a neighborhood socioeconomic index for health inequalities analysis. Int J Equity Health. 2013;12: 21. doi:10.1186/1475-9276-12-21

10. Darling ES. Assessing the Effect of Marine Reserves on Household Food Security in Kenyan Coral Reef Fishing Communities. PLoS One. 2014;9: e113614. doi:10.1371/journal.pone.0113614

11. Cutter SL, Carolina S, Boruff BJ, Shirley WL. Social Vulnerability to Environmental Hazards. Soc Sci Q. 2003;84: 242–261.

12. Nelson R, Kokic P, Crimp S, Meinke H, Howden SM. The vulnerability of Australian rural communities to climate variability and change: Part I—Conceptualising and measuring

vulnerability. Environ Sci Policy. 2010;13: 8–17. doi:10.1016/j.envsci.2009.09.006

13. Tesso G, Emana B, Ketema M. Analysis of vulnerability and resilience to climate change induced shocks in North Shewa, Ethiopia. Agric Sci. 2012;3: 871–888.

14. Jacob S, Weeks P, Blount BG, Jepson M. Exploring fishing dependence in gulf coast communities. Mar Policy. 2010;34: 1307–1314. doi:10.1016/j.marpol.2010.06.003

15. Jacob S, Weeks P, Blount B, Jepson M. Development and evaluation of social indicators of vulnerability and resiliency for fishing communities in the Gulf of Mexico. Mar Policy. 2013;37: 86–95.

16. Kolenikov S, Angeles G. Socioeconomic status measurement with discrete proxy variables : Is principal component analysis a reliable answer? Rev Income Wealth. 2009;55: 128–165.

17. Kolenikov S. The Use of Discrete Data in PCA: Theory, Simulations, and Applications to Socioeconomic Indices. MEASURE Working Paper No. WP-04-85. Chapel Hill: Carolina Population Center, University of North Carolina, USA. 2004.

18. Howe LD, Hargreaves JR, Huttly SRA. Issues in the construction of wealth indices for the measurement of socio-economic position in low-income countries. Emerg Themes Epidemiol. 2008;5: 3. doi:10.1186/1742-7622-5-3

19. Pagès J. Analyse factorielle de données mixtes. Rev Stat appliquée. 2004;52: 93–111.

20. Pagès J. Multiple factor analysis by example using R. CRC Press; 2014.

21. Le S, Josse J, Husson F. FactoMineR : An R Package for Multivariate Analysis. J Stat Softw. 2008;25: 1–18.

22. Husson F, Josse J, Le S, Mazet J. FactoMineR. 2014.

23. Sharker MAY, Nasser M, Abedin J, Arnold BF, Luby SP. The risk of misclassifying subjects within principal component based asset index. Emerg Themes Epidem. 2014;11:1-8.

24. Narain U, Gupta S, van 't Veld K. Poverty and resource dependence in rural India. Ecol Econ. 2008;66: 161–176. doi:10.1016/j.ecolecon.2007.08.021

25. Béné C. Are Fishers Poor or Vulnerable? Assessing Economic Vulnerability in Small-Scale Fishing Communities. J Dev Stud. 2009;45: 911–933. doi:10.1080/00220380902807395

26. Chen C, López-Carr D, Walker BLE. A framework to assess the vulnerability of California commercial sea urchin fishermen to the impact of MPAs under climate change. GeoJournal. 2014;79: 755-773. doi: 10.1007/s10708-014-9543-0.

27. Junio RP, Gonzales AC, Montañ TG. Understanding the social vulnerability of coastal communities. 2015;6. doi:10.7763/IJESD.2015.V6.690

28. Ali ML, Hossain MB, Rokunuzzaman M, Bhadra S. Access to fisheries resources by the poor fishers for income generation and livelihood and their coping strategies during lean and ban fishing period in Bangladesh. Final Report CF # 9/08. Bangladesh Centre for Advanced Studies, Dhaka; 2010.

29. Rahman MA, Emran M, Islam S. Status of hilsa fisheries in Bangladesh. Regional Consultation on Preparation of Management Plan for Hilsa Fisheries. Chittagong, Bangladesh, 7 - 8 February 2010. Chittagong; 2010. Report No.: RC-HF2/5.

30. Harrison M, Baker J, Twinamatsiko M, Milner-Gulland EJ. Profiling unauthorized natural resource users for better targeting of conservation interventions. Conserv Biol. 2015;29: 1636–1646.

31. Ambastha K, Hussain SA, Badola R. Resource dependence and attitudes of local people toward conservation of Kabartal wetland: a case study from the Indo-Gangetic plains.

Wetl Ecol Manag. 2007;15: 287–302. doi:10.1007/s11273-006-9029-z.
