# Supplementary material for: Evaluating the ecological and social targeting of a compensation scheme in Bangladesh
Source: PLoS One. 2018 Jun 13;13(6):e0197809. doi: 10.1371/journal.pone.0197809 (PMC5999081; doi:10.1371/journal.pone.0197809)
Supplement: S4 Appendix — (PDF) [file pone.0197809.s004.pdf]

The respondents had a mean age of 39, household size of 5.6, household dependency ratio of 3.2 dependents per economically active household member, and 2.4 years of education. 16% of respondents were female, and only one of these was a household head. These statistics are similar to that of another recent survey of hilsa fishers carried out in the sanctuary areas [1] – although these authors found the household dependency ratio to be much lower at 1.49. This may be due to differences in how 'economically active' was defined (in this survey respondents were asked to state how many members of the household were earning, rather than using the number of members of working age – of which there may be those who are not working).

The main alternative to fishing was day labour, followed by agriculture (non-livestock), then business (Fig A). 70% of respondents owned homestead land, 30% were landless, and the remainder owned agricultural land. Within this group the mean number of agricultural acres was 0.57. Households with livelihoods other than fishing had significantly more acres of agricultural land than those without (Wilcoxon rank sum test W = 54561.5; $p < 0.05$), indicating that non-livestock farming plays a more prominent role in less fishing-active households. 58% of respondents owned livestock, but ownership was not significantly associated with having other livelihoods ($\chi^2 = 0.09$; df = 1; $p = 0.77$) or with percentage income from fishing (Wilcoxon rank sum test W = 77739; $p = 0.9631$), indicating that owning livestock does not play a strong role in fishing dependence.

68% of respondents owned a boat and there was a significant negative association between boat ownership and having other livelihoods ($\chi^2 = 7.76$; df = 1; $p < 0.01$). As expected, boat owners also had significantly higher proportions of income from fishing (Wilcoxon rank sum test W = 48509; $p < 0.001$). Although the owners of large trawlers are likely to have other sources of income, the small and medium boat owners in this sample appeared to be constrained in their flexibility to engage in other livelihoods by their financial investment. Households with other livelihoods had significantly higher proportions of income from fishing than their other livelihoods (Wilcoxon rank sum test W = 109223; $p < 0.001$).

The income, asset and coping strategy profiles are quite consistent with those found by other authors [1], although they found the proportion who fish illegally to cope during fishing bans to be much lower (27%) than the 40% in this study.

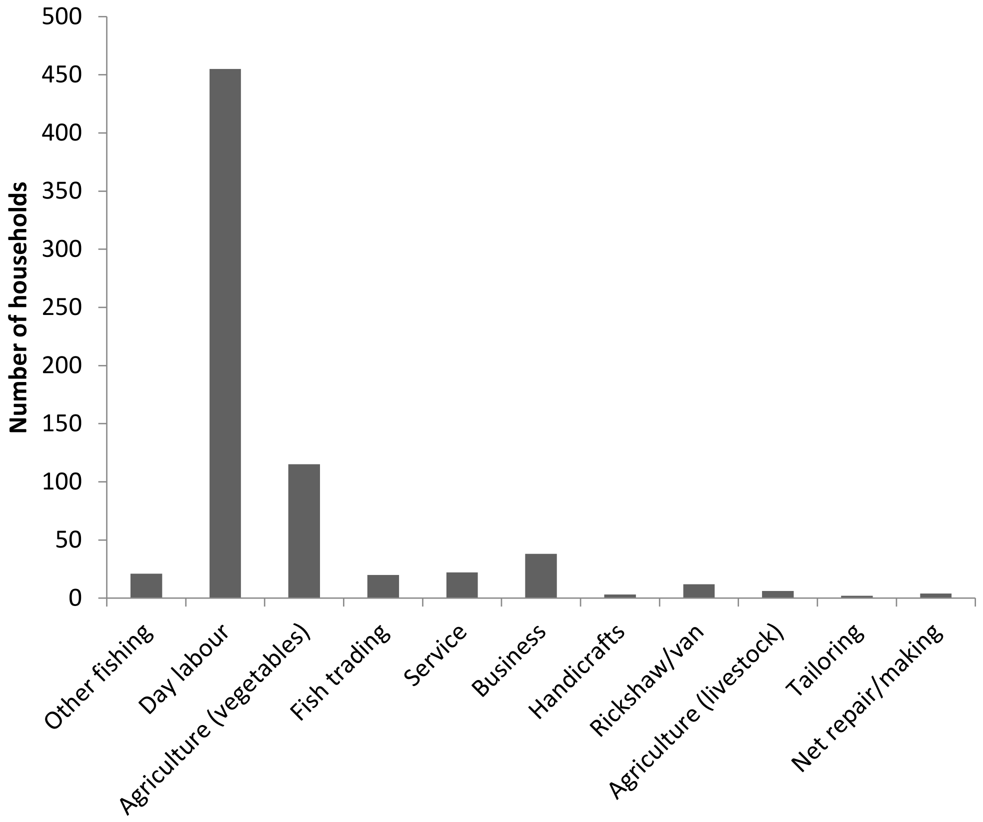

Fig A. Bar plot showing the number of households who participate in various livelihoods. The total household number is greater than the total sample size because some households have more than one livelihood.

References

1. Rahman HZ, Wahab MA, Choudhury LA. Hilsa and hilsa fishermen: exploring conservation-livelihood win-wins. Power and Participation Research Centre: Dhaka, Bangladesh; 2014.
