# Supplementary material for: Evaluating the ecological and social targeting of a compensation scheme in Bangladesh
Source: PLoS One. 2018 Jun 13;13(6):e0197809. doi: 10.1371/journal.pone.0197809 (PMC5999081; doi:10.1371/journal.pone.0197809)
Supplement: S2 Fig — The total percentage is more than 100 because some respondents (n = 799) gave multiple answers. (PDF) [file pone.0197809.s006.pdf]

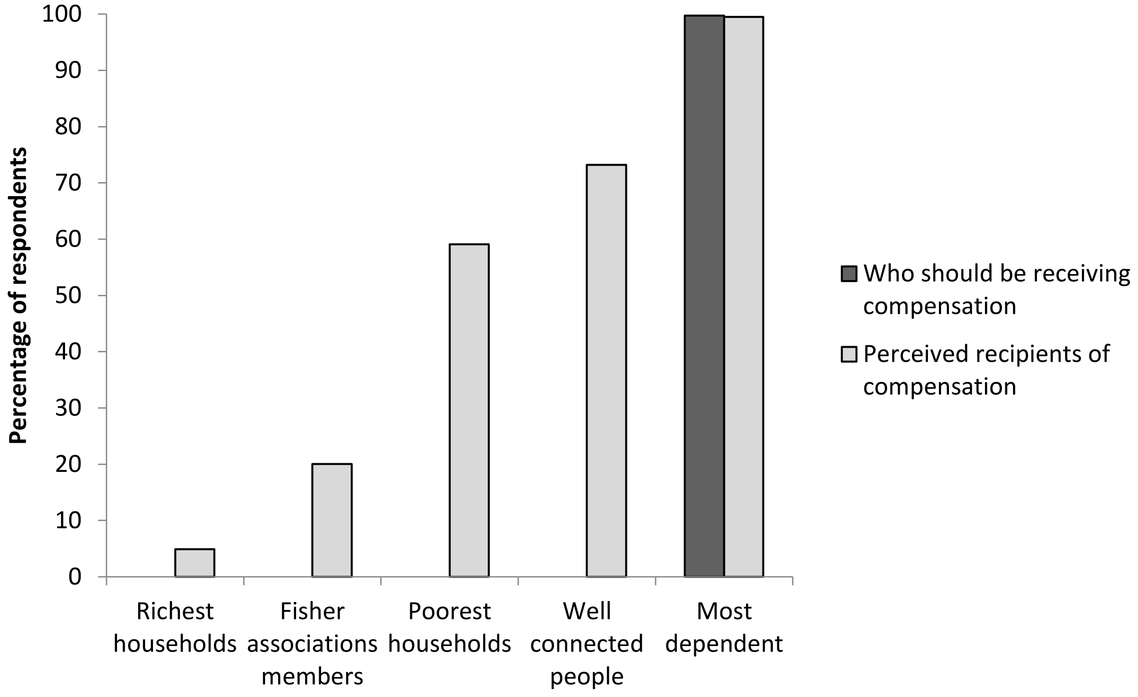

S2 Fig. Groups of people that respondents perceived to be and thought should be receiving compensation. The total percentage is more than 100 because some respondents (*n*=799) gave multiple answers.
