# Supplementary material for: Evaluating the ecological and social targeting of a compensation scheme in Bangladesh
Source: PLoS One. 2018 Jun 13;13(6):e0197809. doi: 10.1371/journal.pone.0197809 (PMC5999081; doi:10.1371/journal.pone.0197809)
Supplement: S3 Fig — The x axes show the effect of living in a particular district (a) or village (b) in terms of the difference in probability of reporting fairness from the intercept. Error bars show the 95% confidence interval based on the conditional variance for each random effect. Village names are prefixed by district. (PDF) [file pone.0197809.s007.pdf]

(a)

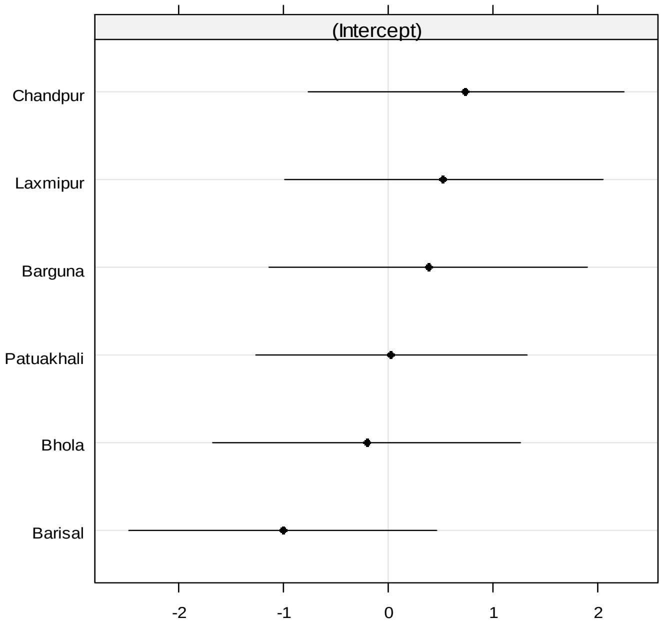

(b)

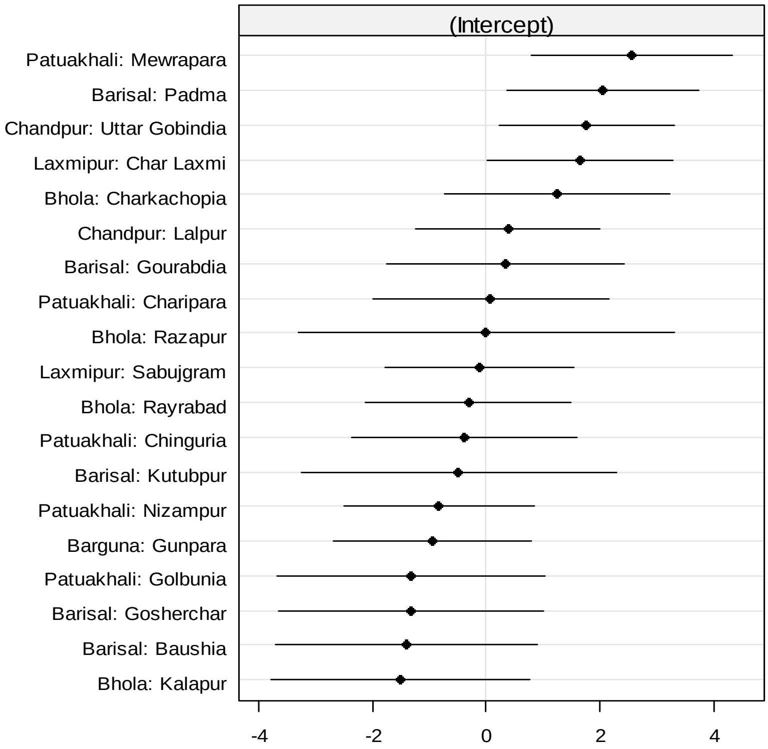

S3 Fig. Best Linear Unbiased Predictors (BLUPs) for the random effects for the probability of reporting fairness of compensation distribution. The x axes show the effect of living in a particular district (a) or village (b) in terms of the difference in probability of reporting fairness from the intercept. Error bars show the 95% confidence interval based on the conditional variance for each random effect. Village names are prefixed by district.
