# Supplementary material for: Evaluating the ecological and social targeting of a compensation scheme in Bangladesh
Source: PLoS One. 2018 Jun 13;13(6):e0197809. doi: 10.1371/journal.pone.0197809 (PMC5999081; doi:10.1371/journal.pone.0197809)
Supplement: S1 Table — Source: DoF 2014. (PDF) [file pone.0197809.s008.pdf]

S1 Table. Distribution of food grain compensation for hilsa fishers in Bangladesh from 2004 to 2014.

Year	Total volume distributed (mt)	No. districts	No. of upazila (sub-districts)	No. of households	Monthly allocation per household (kg)	Duration (months per year)
2004-2005	1,000.00	-	-	33,300	10	3
2005-2006	0	0	0	0	0	0
2006-2007	1,546.00	-	-	103,000	-	1
2007-2008	4,360.00	10	59	145,335	10	3
2008-2009	5,730.08	10	59	143,252	10	3
2009-2010	19,768.60	10	59	164,740	30	4
2010-2011	14,470.64	15	85	186,264	20	4
2011-2012	22,351.68	15	85	186,264	30	4
2012-2013	24,747.48	16	88	206,229	30	4
2013-2014	36,296.32	15	88	224,102	40	4

Source

DoF. Hilsa Village Fishing Statistics. Dhaka: Bangladesh Government, Department of Fisheries; 2014.
