# Supplementary material for: Evaluating the ecological and social targeting of a compensation scheme in Bangladesh
Source: PLoS One. 2018 Jun 13;13(6):e0197809. doi: 10.1371/journal.pone.0197809 (PMC5999081; doi:10.1371/journal.pone.0197809)
Supplement: S2 Table — (PDF) [file pone.0197809.s009.pdf]

S2 Table. Model selection table for GLMM with probability of receiving compensation.

(Intercept)	Fisher association membership	Food Insecurity	Dependency ratio	Household Size	Income	Respondent identity	Sanctuary	Jatka fishing	Fishing dependence	Debt	df	logLik	AICc	delta	weight
0.38789	-0.48528	0.264463	-0.17669	0.29694	NA	NA	NA	NA	NA	NA	7	-403.102	820.3462	0	0.030774
0.388714	-0.4754	NA	-0.16933	0.281568	NA	NA	NA	NA	NA	NA	6	-404.208	820.5226	0.176382	0.028176
0.387466	-0.46356	NA	NA	0.239418	NA	NA	NA	NA	NA	NA	5	-405.302	820.6804	0.334117	0.026039
0.386382	-0.47252	0.252911	NA	0.252417	NA	NA	NA	NA	NA	NA	6	-404.289	820.684	0.337801	0.025991
0.389055	-0.53937	0.255025	NA	0.207568	0.186455	NA	NA	NA	NA	NA	7	-403.42	820.9828	0.636584	0.022385
0.390088	-0.52902	NA	NA	0.194767	0.184156	NA	NA	NA	NA	NA	6	-404.449	821.0054	0.659205	0.022133
0.383961	NA	0.257772	-0.17083	0.288434	NA	NA	NA	NA	NA	NA	6	-404.531	821.1689	0.822684	0.020396
0.384843	NA	NA	-0.16377	0.273609	NA	NA	NA	NA	NA	NA	5	-405.582	821.2405	0.894298	0.019678
0.383709	NA	NA	NA	0.232789	NA	NA	NA	NA	NA	NA	4	-406.608	821.2676	0.921325	0.019414
0.382571	NA	0.246746	NA	0.245335	NA	NA	NA	NA	NA	NA	5	-405.643	821.3633	1.017099	0.018506
0.389623	-0.53028	0.264024	-0.14642	0.256244	0.137058	NA	NA	NA	NA	NA	8	-402.668	821.5194	1.173172	0.017117
0.387556	-0.53919	NA	NA	NA	0.24989	NA	NA	NA	NA	NA	5	-405.771	821.6186	1.272394	0.016289
0.390487	-0.52023	NA	-0.13894	0.240601	0.137352	NA	NA	NA	NA	NA	7	-403.769	821.6811	1.334882	0.015788
0.386736	-0.54937	0.23183	NA	NA	0.25581	NA	NA	NA	NA	NA	6	-404.908	821.9222	1.575988	0.013995
0.389379	-0.49125	0.274844	-0.17772	0.2984	NA	0.077311	NA	NA	NA	NA	8	-402.946	822.0763	1.73008	0.012957
0.385056	NA	NA	NA	0.199345	0.136348	NA	NA	NA	NA	NA	5	-406.104	822.2844	1.938169	0.011677
0.370026	-0.48533	0.264904	-0.17656	0.297223	NA	NA	-0.16526	NA	NA	NA	8	-403.091	822.3666	2.020401	0.011206
0.387903	-0.48575	0.264321	-0.17591	0.296071	NA	NA	NA	0.013859	NA	NA	8	-403.097	822.3778	2.031567	0.011144
0.387493	-0.48638	0.262571	-0.17608	0.296617	NA	NA	NA	NA	-0.0118	NA	8	-403.097	822.3779	2.031696	0.011143
0.383928	NA	0.247282	NA	0.211894	0.13716	NA	NA	NA	NA	NA	6	-405.136	822.3783	2.032055	0.011141
0.387933	-0.48509	0.265133	-0.17685	0.29662	NA	NA	NA	NA	NA	0.006047	8	-403.101	822.3865	2.040238	0.011096
0.389737	-0.47931	NA	-0.16996	0.282263	NA	0.054855	NA	NA	NA	NA	7	-404.129	822.4002	2.054	0.011019
0.387677	-0.47827	0.262957	NA	0.253538	NA	0.07354	NA	NA	NA	NA	7	-404.147	822.4372	2.090979	0.010818
0.387441	-0.47859	NA	-0.16777	0.280925	NA	NA	NA	NA	-0.03254	NA	7	-404.173	822.4882	2.141956	0.010545
0.388495	-0.4771	NA	-0.16839	0.284208	NA	NA	NA	NA	NA	-0.0443	7	-404.191	822.5239	2.177708	0.010359
0.373529	-0.47542	NA	-0.16921	0.281791	NA	NA	-0.14105	NA	NA	NA	7	-404.201	822.5441	2.197866	0.010255
0.388784	-0.47595	NA	-0.16844	0.280594	NA	NA	NA	0.015833	NA	NA	7	-404.202	822.546	2.199795	0.010245
0.388388	-0.46732	NA	NA	0.239902	NA	0.051782	NA	NA	NA	NA	6	-405.231	822.5693	2.223062	0.010126

0.385885	-0.46791	NA	NA	0.239211	NA	NA	NA	NA	-0.04209	NA	6	-405.243	822.5938	2.247578	0.010003
0.38716	-0.46599	NA	NA	0.24326	NA	NA	NA	NA	NA	-0.05949	6	-405.271	822.6483	2.302021	0.009734
0.387644	-0.46467	NA	NA	0.237975	NA	NA	NA	0.030435	NA	NA	6	-405.279	822.6649	2.318697	0.009653
0.386404	-0.47359	0.252689	NA	0.251021	NA	NA	NA	0.029167	NA	NA	7	-404.267	822.6777	2.331489	0.009592
0.385561	-0.47475	0.249303	NA	0.252164	NA	NA	NA	NA	-0.02282	NA	7	-404.271	822.6857	2.339427	0.009554
0.371022	-0.46359	NA	NA	0.23969	NA	NA	-0.15265	NA	NA	NA	6	-405.294	822.6941	2.347905	0.009513
0.367282	-0.4726	0.253392	NA	0.252761	NA	NA	-0.17624	NA	NA	NA	7	-404.277	822.6965	2.350277	0.009502
0.386318	-0.47298	0.251568	NA	0.253161	NA	NA	NA	NA	NA	-0.01257	7	-404.287	822.7175	2.371284	0.009403
0.384698	NA	0.256844	-0.15089	0.261582	0.089394	NA	NA	NA	NA	NA	7	-404.33	822.8037	2.457447	0.009006
0.390158	-0.54276	0.263496	NA	0.20956	0.182509	0.062949	NA	NA	NA	NA	8	-403.317	822.8182	2.471942	0.008941
0.385635	NA	NA	-0.14346	0.246199	0.090977	NA	NA	NA	NA	NA	6	-405.373	822.8538	2.507518	0.008784
0.389392	-0.54343	0.254762	NA	0.203244	0.193742	NA	NA	0.053787	NA	NA	8	-403.35	822.8834	2.537134	0.008655
0.390691	-0.53315	NA	NA	0.190418	0.191471	NA	NA	0.054576	NA	NA	7	-404.377	822.896	2.549775	0.0086
0.390845	-0.53102	NA	NA	0.195839	0.181569	0.041315	NA	NA	NA	NA	7	-404.404	822.9517	2.605506	0.008364
0.389753	-0.53217	NA	NA	0.198911	0.185499	NA	NA	NA	NA	-0.06944	7	-404.406	822.9555	2.609248	0.008348
0.385217	NA	0.266915	-0.17172	0.28963	NA	0.06845	NA	NA	NA	NA	7	-404.408	822.9597	2.613443	0.008331
0.389963	-0.53937	0.258764	NA	0.206056	0.193783	NA	NA	NA	0.022945	NA	8	-403.405	822.9933	2.647017	0.008192
0.369474	-0.53947	0.255523	NA	0.207902	0.186538	NA	-0.18068	NA	NA	NA	8	-403.407	822.9989	2.65262	0.008169
0.388922	-0.54028	0.25262	NA	0.208782	0.186868	NA	NA	NA	NA	-0.0224	8	-403.416	823.0157	2.669437	0.008101
0.383205	-0.44016	NA	NA	NA	NA	NA	NA	NA	NA	NA	4	-407.483	823.0176	2.671408	0.008093
0.37326	-0.52909	NA	NA	0.195036	0.184217	NA	-0.15648	NA	NA	NA	7	-404.44	823.0232	2.676986	0.00807
0.390112	-0.52903	NA	NA	0.194725	0.184372	NA	NA	NA	0.00069	NA	7	-404.449	823.0413	2.69506	0.007998
0.382335	NA	NA	NA	NA	0.199784	NA	NA	NA	NA	NA	4	-407.496	823.0438	2.697582	0.007987
0.385722	NA	NA	-0.16429	0.274143	NA	0.046871	NA	NA	NA	NA	6	-405.524	823.1551	2.808852	0.007555
0.383706	NA	0.255559	NA	0.246233	NA	0.064905	NA	NA	NA	NA	6	-405.533	823.1727	2.826436	0.007489
0.365906	NA	0.258209	-0.17071	0.288734	NA	NA	-0.1668	NA	NA	NA	7	-404.521	823.1842	2.837921	0.007446
0.384488	NA	NA	NA	0.233137	NA	0.043994	NA	NA	NA	NA	5	-406.557	823.1903	2.844051	0.007423
0.384092	NA	0.259821	-0.1713	0.287475	NA	NA	NA	NA	NA	0.018537	7	-404.528	823.198	2.851752	0.007395
0.383955	NA	0.25766	-0.17028	0.287839	NA	NA	NA	0.009509	NA	NA	7	-404.529	823.2003	2.854026	0.007386
0.383833	NA	0.257212	-0.17065	0.288328	NA	NA	NA	NA	-0.00339	NA	7	-404.531	823.2039	2.857675	0.007373
0.382418	NA	NA	NA	0.23252	NA	NA	NA	NA	-0.03315	NA	5	-406.572	823.2195	2.873259	0.007316

0.383875	NA	NA	-0.16261	0.273061	NA	NA	NA	NA	-0.02384	NA	6	-405.563	823.2329	2.886673	0.007267
0.384667	NA	NA	-0.1631	0.275376	NA	NA	NA	NA	NA	-0.0307	6	-405.574	823.2547	2.908491	0.007188
0.383424	NA	NA	NA	0.235651	NA	NA	NA	NA	NA	-0.04538	5	-406.59	823.2564	2.910194	0.007182
0.36941	NA	NA	-0.16365	0.273836	NA	NA	-0.14328	NA	NA	NA	6	-405.575	823.2566	2.910379	0.007181
0.383821	NA	NA	NA	0.231558	NA	NA	NA	0.025887	NA	NA	5	-406.592	823.2594	2.913174	0.007171
0.384878	NA	NA	-0.16311	0.272899	NA	NA	NA	0.011475	NA	NA	6	-405.579	823.2646	2.918364	0.007153
0.367107	NA	NA	NA	0.233064	NA	NA	-0.1541	NA	NA	NA	5	-406.6	823.2762	2.929915	0.007111
0.387215	-0.53542	NA	-0.06881	NA	0.234241	NA	NA	NA	NA	NA	6	-405.587	823.2808	2.934549	0.007095
0.390915	-0.53381	0.273242	-0.14838	0.259093	0.13201	0.068831	NA	NA	NA	NA	9	-402.545	823.3208	2.974557	0.006954
0.382585	NA	0.246555	NA	0.244153	NA	NA	NA	0.024619	NA	NA	6	-405.628	823.3637	3.017458	0.006807
0.388643	-0.54492	NA	NA	NA	0.258524	NA	NA	0.076017	NA	NA	6	-405.628	823.364	3.017735	0.006806
0.3634	NA	0.247219	NA	0.245679	NA	NA	-0.177	NA	NA	NA	6	-405.632	823.3709	3.024655	0.006782
0.379845	NA	NA	NA	NA	NA	NA	NA	NA	NA	NA	3	-408.673	823.3766	3.030384	0.006763
0.382061	NA	0.24448	NA	0.245132	NA	NA	NA	NA	-0.01411	NA	6	-405.637	823.3806	3.034364	0.00675
0.382585	NA	0.246811	NA	0.245304	NA	NA	NA	NA	NA	0.000567	6	-405.643	823.394	3.047771	0.006704
0.382405	-0.44694	0.221894	NA	NA	NA	NA	NA	NA	NA	NA	5	-406.688	823.453	3.106775	0.00651
0.381456	NA	0.223271	NA	NA	0.203952	NA	NA	NA	NA	NA	5	-406.695	823.4655	3.119291	0.006469
0.389778	-0.5331	0.26365	-0.14324	0.252416	0.142716	NA	NA	0.034669	NA	NA	9	-402.639	823.5079	3.161693	0.006333
0.371155	-0.53038	0.2645	-0.14626	0.256518	0.137194	NA	-0.17053	NA	NA	NA	9	-402.657	823.5434	3.197206	0.006222
0.390382	-0.53021	0.267166	-0.14605	0.254934	0.143253	NA	NA	NA	0.019544	NA	9	-402.657	823.5435	3.197267	0.006222
0.386475	-0.54544	0.233726	-0.07162	NA	0.239451	NA	NA	NA	NA	NA	7	-404.708	823.5598	3.213598	0.006171
0.389586	-0.53046	0.263563	-0.14628	0.256432	0.137188	NA	NA	NA	NA	-0.00427	9	-402.668	823.5655	3.219296	0.006153
0.38821	-0.54075	NA	NA	NA	0.247949	0.03425	NA	NA	NA	NA	6	-405.74	823.5875	3.241224	0.006086
0.391335	-0.52239	NA	-0.14017	0.242228	0.133984	0.046409	NA	NA	NA	NA	8	-403.713	823.6097	3.263437	0.006019
0.388266	-0.53915	NA	NA	NA	0.254945	NA	NA	NA	0.01672	NA	6	-405.763	823.6326	3.286391	0.00595
0.373788	-0.53924	NA	NA	NA	0.25001	NA	-0.12772	NA	NA	NA	6	-405.765	823.6377	3.291476	0.005935
0.387319	-0.54031	NA	NA	NA	0.250875	NA	NA	NA	NA	-0.02493	6	-405.766	823.6384	3.292209	0.005933
0.390807	-0.52319	NA	-0.13562	0.236613	0.143247	NA	NA	0.036578	NA	NA	8	-403.737	823.6576	3.311367	0.005877
0.387554	-0.5552	0.232167	NA	NA	0.264662	NA	NA	0.076662	NA	NA	7	-404.763	823.6688	3.322538	0.005844
0.390207	-0.52277	NA	-0.13742	0.243368	0.138911	NA	NA	NA	NA	-0.05441	8	-403.743	823.6698	3.323526	0.005841
0.374747	-0.52029	NA	-0.13879	0.240807	0.137452	NA	-0.14598	NA	NA	NA	8	-403.761	823.7065	3.360313	0.005735

0.390346	-0.52026	NA	-0.13901	0.240831	0.136372	NA	NA	NA	-0.00309	NA	8	-403.769	823.7215	3.375282	0.005692
0.387729	-0.55213	0.238954	NA	NA	0.252865	0.053748	NA	NA	NA	NA	7	-404.832	823.8074	3.461122	0.005453
0.388293	-0.54964	0.238562	NA	NA	0.26774	NA	NA	NA	0.038661	NA	7	-404.863	823.8695	3.523269	0.005286
0.379032	NA	0.216895	NA	NA	NA	NA	NA	NA	NA	NA	4	-407.914	823.8781	3.53191	0.005263
0.37073	-0.54948	0.232208	NA	NA	0.255972	NA	-0.14783	NA	NA	NA	7	-404.899	823.9418	3.595548	0.005098
0.386923	-0.54854	0.234188	NA	NA	0.255044	NA	NA	NA	NA	0.020814	7	-404.903	823.9496	3.603364	0.005078
0.371779	-0.49128	0.275265	-0.17759	0.298685	NA	0.077186	-0.16285	NA	NA	NA	9	-402.936	824.1027	3.756505	0.004704
0.389381	-0.49177	0.274703	-0.17687	0.297481	NA	0.077497	NA	0.014922	NA	NA	9	-402.941	824.1116	3.765387	0.004683
0.389012	-0.49213	0.273204	-0.17719	0.298117	NA	0.077006	NA	NA	-0.00981	NA	9	-402.943	824.1161	3.769833	0.004673
0.389455	-0.49084	0.276282	-0.17805	0.297741	NA	0.077679	NA	NA	NA	0.012619	9	-402.945	824.1194	3.773116	0.004665
0.382975	-0.44539	NA	-0.1045	NA	NA	NA	NA	NA	NA	NA	5	-407.041	824.158	3.811813	0.004576
0.385397	NA	NA	NA	0.195929	0.141807	NA	NA	0.044027	NA	NA	6	-406.056	824.2195	3.873231	0.004437
0.385693	NA	NA	NA	0.200224	0.134085	0.035502	NA	NA	NA	NA	6	-406.071	824.2486	3.902394	0.004373
0.384916	NA	0.254869	NA	0.213616	0.133532	0.056299	NA	NA	NA	NA	7	-405.053	824.2487	3.902456	0.004373
0.384696	NA	NA	NA	0.202408	0.137158	NA	NA	NA	NA	-0.05174	6	-406.08	824.2672	3.920916	0.004333
0.368133	NA	NA	NA	0.19961	0.136405	NA	-0.15728	NA	NA	NA	6	-406.095	824.2972	3.950985	0.004268
0.385086	NA	NA	NA	0.199293	0.13657	NA	NA	NA	0.000738	NA	6	-406.104	824.3151	3.968838	0.00423
0.384087	NA	0.247002	NA	0.208534	0.142542	NA	NA	0.042997	NA	NA	7	-405.09	824.3233	3.977087	0.004213
