# Supplementary material for: Evaluating the ecological and social targeting of a compensation scheme in Bangladesh
Source: PLoS One. 2018 Jun 13;13(6):e0197809. doi: 10.1371/journal.pone.0197809 (PMC5999081; doi:10.1371/journal.pone.0197809)
Supplement: S3 Table — (PDF) [file pone.0197809.s010.pdf]

Table S3. Model selection table for GLMM with probability of receiving compensation, excluding Chandpur.

Intercept	Fishing association member	Food insecurity	Sanctuary	Dependency ratio	Household size	Household income	Respondent identity	Jatka fishing	Loan	Fishing dependence	df	logLik	AICc	delta	weight
-0.1922	-0.4392	0.2741	-0.9217	-0.1725	0.2831	NA	NA	NA	NA	NA	8	-392.9250	802.0775	0.0000	0.0318
-0.1956	-0.4285	0.2627	-0.9361	NA	0.2398	NA	NA	NA	NA	NA	7	-394.1135	802.4036	0.3261	0.0270
-0.1908	-0.4245	NA	-0.8933	-0.1650	0.2664	NA	NA	NA	NA	NA	7	-394.1282	802.4330	0.3555	0.0266
-0.2018	NA	0.2647	-0.9395	-0.1679	0.2778	NA	NA	NA	NA	NA	7	-394.1435	802.4636	0.3862	0.0262
-0.1940	-0.4148	NA	-0.9085	NA	0.2255	NA	NA	NA	NA	NA	6	-395.2203	802.5729	0.4955	0.0248
-0.2000	NA	NA	-0.9115	-0.1609	0.2617	NA	NA	NA	NA	NA	6	-395.2678	802.6680	0.5905	0.0237
-0.2049	NA	0.2539	-0.9529	NA	0.2355	NA	NA	NA	NA	NA	6	-395.2721	802.6765	0.5991	0.0236
-0.2029	NA	NA	-0.9257	NA	0.2218	NA	NA	NA	NA	NA	5	-396.3077	802.7097	0.6323	0.0232
-0.1929	-0.4914	0.2650	-0.9385	NA	0.1961	0.1499	NA	NA	NA	NA	8	-393.2598	802.7470	0.6695	0.0227
-0.1914	-0.4763	NA	-0.9105	NA	0.1820	0.1477	NA	NA	NA	NA	7	-394.3858	802.9482	0.8707	0.0206
-0.1910	-0.4814	0.2738	-0.9261	-0.1432	0.2437	0.1095	NA	NA	NA	NA	9	-392.5045	803.2937	1.2163	0.0173
-0.1876	-0.4905	NA	-0.8861	NA	NA	0.1997	NA	NA	NA	NA	6	-395.6109	803.3541	1.2766	0.0168
-0.1884	-0.5055	0.2408	-0.9097	NA	NA	0.2053	NA	NA	NA	NA	7	-394.6668	803.5102	1.4328	0.0155
-0.1896	-0.4666	NA	-0.8976	-0.1357	0.2268	0.1095	NA	NA	NA	NA	8	-393.7047	803.6368	1.5594	0.0146
-0.1915	-0.4472	0.2861	-0.9254	-0.1738	0.2852	NA	0.0963	NA	NA	NA	9	-392.6833	803.6513	1.5738	0.0145
-0.2038	NA	0.2544	-0.9564	NA	0.2020	0.1140	NA	NA	NA	NA	7	-394.7425	803.6617	1.5843	0.0144
-0.2019	NA	NA	-0.9290	NA	0.1883	0.1134	NA	NA	NA	NA	6	-395.7818	803.6959	1.6185	0.0142
-0.1949	-0.4364	0.2745	-0.9399	NA	0.2415	NA	0.0921	NA	NA	NA	8	-393.8916	804.0107	1.9333	0.0121
-0.2016	NA	0.2638	-0.9436	-0.1477	0.2507	0.0748	NA	NA	NA	NA	8	-393.9323	804.0921	2.0146	0.0116
-0.1926	-0.4398	0.2736	-0.9287	-0.1737	0.2850	NA	NA	-0.0239	NA	NA	9	-392.9100	804.1049	2.0274	0.0115
-0.2013	NA	0.2755	-0.9432	-0.1691	0.2796	NA	0.0868	NA	NA	NA	8	-393.9458	804.1191	2.0416	0.0115
-0.1920	-0.4383	0.2771	-0.9231	-0.1731	0.2816	NA	NA	NA	0.0287	NA	9	-392.9175	804.1197	2.0423	0.0115
-0.1923	-0.4398	0.2731	-0.9219	-0.1722	0.2830	NA	NA	NA	NA	-0.0050	9	-392.9240	804.1328	2.0554	0.0114
-0.1903	-0.4300	NA	-0.8953	-0.1660	0.2675	NA	0.0739	NA	NA	NA	8	-393.9848	804.1971	2.1197	0.0110
-0.1998	NA	NA	-0.9156	-0.1403	0.2341	0.0761	NA	NA	NA	NA	7	-395.0479	804.2725	2.1951	0.0106
-0.1986	NA	NA	-0.9047	NA	NA	0.1640	NA	NA	NA	NA	5	-397.1003	804.2949	2.2174	0.0105
-0.1934	-0.4202	NA	-0.9106	NA	0.2263	NA	0.0702	NA	NA	NA	7	-395.0901	804.3569	2.2795	0.0102
-0.2043	NA	0.2644	-0.9566	NA	0.2369	NA	0.0828	NA	NA	NA	7	-395.0916	804.3599	2.2824	0.0102
-0.1913	-0.4284	NA	-0.8948	-0.1638	0.2662	NA	NA	NA	NA	-0.0271	8	-394.1019	804.4312	2.3537	0.0098
-0.1958	-0.4307	0.2601	-0.9366	NA	0.2398	NA	NA	NA	NA	-0.0155	8	-394.1049	804.4373	2.3598	0.0098
-0.1912	-0.4252	NA	-0.9011	-0.1665	0.2685	NA	NA	-0.0266	NA	NA	8	-394.1095	804.4465	2.3690	0.0097
-0.1957	-0.4287	0.2625	-0.9390	NA	0.2404	NA	NA	-0.0098	NA	NA	8	-394.1110	804.4494	2.3719	0.0097
-0.1955	-0.4281	0.2639	-0.9367	NA	0.2391	NA	NA	NA	0.0114	NA	8	-394.1122	804.4519	2.3745	0.0097
-0.1924	-0.4964	0.2754	-0.9418	NA	0.1989	0.1457	0.0817	NA	NA	NA	9	-393.0860	804.4569	2.3794	0.0097
-0.1910	-0.4252	NA	-0.8924	-0.1646	0.2677	NA	NA	NA	-0.0217	NA	8	-394.1239	804.4754	2.3979	0.0096
-0.1996	NA	NA	-0.9134	-0.1617	0.2627	NA	0.0657	NA	NA	NA	7	-395.1536	804.4839	2.4064	0.0095
-0.2014	NA	0.2688	-0.9415	-0.1687	0.2758	NA	NA	NA	0.0385	NA	8	-394.1297	804.4868	2.4093	0.0095
-0.2021	NA	0.2644	-0.9456	-0.1690	0.2794	NA	NA	-0.0207	NA	NA	8	-394.1320	804.4915	2.4140	0.0095
-0.2017	NA	0.2658	-0.9392	-0.1682	0.2779	NA	NA	NA	NA	0.0058	8	-394.1425	804.5124	2.4349	0.0094
-0.1945	-0.4202	NA	-0.9104	NA	0.2257	NA	NA	NA	NA	-0.0361	7	-395.1739	804.5245	2.4470	0.0094
-0.2024	NA	NA	-0.9277	NA	0.2224	NA	0.0623	NA	NA	NA	6	-396.2048	804.5418	2.4643	0.0093
-0.1943	-0.4163	NA	-0.9069	NA	0.2278	NA	NA	NA	-0.0359	NA	7	-395.2087	804.5940	2.5165	0.0090
-0.1941	-0.4151	NA	-0.9123	NA	0.2263	NA	NA	-0.0128	NA	NA	7	-395.2160	804.6087	2.5312	0.0090
-0.1999	NA	0.2289	-0.9276	NA	NA	0.1677	NA	NA	NA	NA	6	-396.2453	804.6229	2.5454	0.0089
-0.1903	-0.3998	NA	-0.8756	NA	NA	NA	NA	NA	NA	NA	5	-397.2766	804.6475	2.5700	0.0088
-0.1994	NA	NA	-0.8932	NA	NA	NA	NA	NA	NA	NA	4	-398.2974	804.6576	2.5801	0.0088
-0.2004	NA	NA	-0.9184	-0.1622	0.2636	NA	NA	-0.0236	NA	NA	7	-395.2529	804.6825	2.6050	0.0086
-0.2004	NA	NA	-0.9125	-0.1601	0.2615	NA	NA	NA	NA	-0.0160	7	-395.2585	804.6936	2.6161	0.0086
-0.2034	NA	NA	-0.9272	NA	0.2218	NA	NA	NA	NA	-0.0249	6	-396.2851	804.7026	2.6251	0.0086
-0.2001	NA	NA	-0.9111	-0.1607	0.2623	NA	NA	NA	-0.0103	NA	7	-395.2669	804.7105	2.6330	0.0085
-0.2047	NA	0.2561	-0.9540	NA	0.2342	NA	NA	NA	0.0217	NA	7	-395.2677	804.7120	2.6346	0.0085
-0.2050	NA	0.2538	-0.9550	NA	0.2359	NA	NA	-0.0070	NA	NA	7	-395.2708	804.7183	2.6408	0.0085
-0.2049	NA	0.2531	-0.9531	NA	0.2355	NA	NA	NA	NA	-0.0046	7	-395.2713	804.7192	2.6418	0.0085
-0.2031	NA	NA	-0.9247	NA	0.2233	NA	NA	NA	-0.0241	NA	6	-396.3024	804.7372	2.6597	0.0084
-0.2030	NA	NA	-0.9287	NA	0.2225	NA	NA	-0.0102	NA	NA	6	-396.3049	804.7421	2.6646	0.0084

-0.1924	-0.4900	0.2697	-0.9377	NA	0.1942	0.1566	NA	NA	NA	0.0263	9	-393.2378	804.7605	2.6830	0.0083
-0.1927	-0.4915	0.2653	-0.9353	NA	0.1950	0.1510	NA	0.0110	NA	NA	9	-393.2565	804.7978	2.7204	0.0082
-0.1929	-0.4912	0.2652	-0.9387	NA	0.1960	0.1499	NA	NA	0.0013	NA	9	-393.2598	804.8044	2.7270	0.0081
-0.1910	-0.4795	NA	-0.9121	NA	0.1837	0.1446	0.0600	NA	NA	NA	8	-394.2914	804.8103	2.7328	0.0081
-0.1904	-0.4866	0.2849	-0.9293	-0.1457	0.2476	0.1042	0.0881	NA	NA	NA	10	-392.3037	804.9561	2.8787	0.0075
-0.1913	-0.4111	0.2299	-0.8977	NA	NA	NA	NA	NA	NA	NA	6	-396.4121	804.9565	2.8791	0.0075
-0.1918	-0.4783	NA	-0.9085	NA	0.1848	0.1485	NA	NA	-0.0462	NA	8	-394.3664	804.9603	2.8828	0.0075
-0.1913	-0.4764	NA	-0.9083	NA	0.1814	0.1485	NA	0.0074	NA	NA	8	-394.3843	804.9960	2.9186	0.0074
-0.1913	-0.4761	NA	-0.9104	NA	0.1818	0.1484	NA	NA	NA	0.0028	8	-394.3856	804.9987	2.9213	0.0074
-0.1864	-0.4869	NA	-0.8768	-0.0687	NA	0.1867	NA	NA	NA	NA	7	-395.4177	805.0120	2.9346	0.0073
-0.2006	NA	0.2220	-0.9151	NA	NA	NA	NA	NA	NA	NA	5	-397.4904	805.0752	2.9977	0.0071
-0.1872	-0.5017	0.2424	-0.9003	-0.0711	NA	0.1918	NA	NA	NA	NA	8	-394.4603	805.1481	3.0706	0.0068
-0.1872	-0.4933	NA	-0.8874	NA	NA	0.1973	0.0524	NA	NA	NA	7	-395.5388	805.2543	3.1768	0.0065
-0.1879	-0.5100	0.2496	-0.9124	NA	NA	0.2022	0.0717	NA	NA	NA	8	-394.5324	805.2923	3.2149	0.0064
-0.1905	-0.4802	0.2779	-0.9254	-0.1428	0.2420	0.1154	NA	NA	NA	0.0234	10	-392.4871	805.3229	3.2455	0.0063
-0.1871	-0.4908	NA	-0.8772	NA	NA	0.2022	NA	0.0314	NA	NA	7	-395.5846	805.3459	3.2685	0.0062
-0.1908	-0.4807	0.2757	-0.9270	-0.1437	0.2429	0.1090	NA	NA	0.0184	NA	10	-392.5013	805.3512	3.2737	0.0062
-0.1911	-0.4813	0.2737	-0.9280	-0.1437	0.2445	0.1086	NA	-0.0067	NA	NA	10	-392.5033	805.3553	3.2778	0.0062
-0.1872	-0.4895	NA	-0.8855	NA	NA	0.2042	NA	NA	NA	0.0189	7	-395.5993	805.3753	3.2979	0.0061
-0.1876	-0.4907	NA	-0.8859	NA	NA	0.1998	NA	NA	-0.0031	NA	7	-395.6108	805.3983	3.3209	0.0060
-0.2033	NA	0.2637	-0.9596	NA	0.2045	0.1101	0.0741	NA	NA	NA	8	-394.5989	805.4253	3.3478	0.0060
-0.1877	-0.5037	0.2487	-0.9091	NA	NA	0.2155	NA	NA	NA	0.0420	8	-394.6099	805.4474	3.3699	0.0059
-0.1892	-0.4699	NA	-0.8992	-0.1375	0.2292	0.1056	0.0656	NA	NA	NA	9	-393.5924	805.4696	3.3921	0.0058
-0.1879	-0.5058	0.2420	-0.8995	NA	NA	0.2082	NA	0.0363	NA	NA	8	-394.6315	805.4904	3.4130	0.0058
-0.1882	-0.5039	0.2455	-0.9124	NA	NA	0.2039	NA	NA	0.0433	NA	8	-394.6493	805.5261	3.4486	0.0057
-0.2015	NA	NA	-0.9306	NA	0.1898	0.1106	0.0536	NA	NA	NA	7	-395.7059	805.5884	3.5109	0.0055
-0.2031	NA	0.2598	-0.9555	NA	0.2000	0.1217	NA	NA	NA	0.0303	8	-394.7133	805.6540	3.5766	0.0053
-0.1899	-0.4680	NA	-0.8963	-0.1348	0.2284	0.1103	NA	NA	-0.0319	NA	9	-393.6955	805.6758	3.5983	0.0053
-0.1897	-0.4664	NA	-0.9004	-0.1365	0.2280	0.1083	NA	-0.0095	NA	NA	9	-393.7024	805.6895	3.6121	0.0052
-0.1918	-0.4479	0.2857	-0.9318	-0.1750	0.2869	NA	0.0959	-0.0219	NA	NA	10	-392.6705	805.6897	3.6122	0.0052
-0.1912	-0.4463	0.2901	-0.9274	-0.1747	0.2833	NA	0.0974	NA	0.0365	NA	10	-392.6709	805.6904	3.6130	0.0052
-0.1896	-0.4665	NA	-0.8977	-0.1357	0.2268	0.1093	NA	NA	NA	-0.0005	9	-393.7046	805.6941	3.6166	0.0052
-0.2036	NA	0.2546	-0.9536	NA	0.2011	0.1150	NA	0.0096	NA	NA	8	-394.7401	805.7076	3.6301	0.0052
-0.2036	NA	0.2559	-0.9572	NA	0.2013	0.1138	NA	NA	0.0146	NA	8	-394.7405	805.7085	3.6310	0.0052
-0.1916	-0.4476	0.2858	-0.9255	-0.1737	0.2851	NA	0.0962	NA	NA	-0.0023	10	-392.6830	805.7147	3.6372	0.0052
-0.2022	NA	NA	-0.9278	NA	0.1901	0.1138	NA	NA	-0.0310	NA	7	-395.7730	805.7227	3.6453	0.0051
-0.2017	NA	NA	-0.9287	NA	0.1877	0.1153	NA	NA	NA	0.0076	7	-395.7800	805.7367	3.6593	0.0051
-0.2018	NA	NA	-0.9272	NA	0.1877	0.1140	NA	0.0061	NA	NA	7	-395.7808	805.7383	3.6608	0.0051
-0.1882	-0.4046	NA	-0.8625	-0.1029	NA	NA	NA	NA	NA	NA	6	-396.8256	805.7834	3.7060	0.0050
-0.2011	NA	0.2738	-0.9467	-0.1501	0.2543	0.0698	0.0807	NA	NA	NA	9	-393.7624	805.8097	3.7322	0.0049
-0.1975	NA	NA	-0.8809	-0.0997	NA	NA	NA	NA	NA	NA	5	-397.8730	805.8403	3.7629	0.0048
-0.1973	NA	NA	-0.8950	-0.0718	NA	0.1512	NA	NA	NA	NA	6	-396.8881	805.9085	3.8310	0.0047
-0.1891	-0.4163	0.2335	-0.8847	-0.1062	NA	NA	NA	NA	NA	NA	7	-395.9329	806.0424	3.9649	0.0044
-0.1951	-0.4382	0.2722	-0.9403	NA	0.2414	NA	0.0917	NA	NA	-0.0133	9	-393.8852	806.0553	3.9778	0.0044
-0.1947	-0.4358	0.2765	-0.9410	NA	0.2404	NA	0.0926	NA	0.0185	NA	9	-393.8884	806.0616	3.9841	0.0043
-0.1950	-0.4366	0.2743	-0.9422	NA	0.2420	NA	0.0919	-0.0077	NA	NA	9	-393.8901	806.0649	3.9875	0.0043
