# Supplementary material for: Evaluating the ecological and social targeting of a compensation scheme in Bangladesh
Source: PLoS One. 2018 Jun 13;13(6):e0197809. doi: 10.1371/journal.pone.0197809 (PMC5999081; doi:10.1371/journal.pone.0197809)
Supplement: S4 Table — Results for GLMMs of probability of (a) receiving compensation; and (b) perceiving fair compensation distribution–excluding Chandpur district. (DOCX) [file pone.0197809.s011.docx]

**S4 Table. Results for GLMMs of probability of (a) receiving compensation; and (b) perceiving fair compensation distribution – excluding Chandpur district.**

|  | 1. **Probability of receiving compensation** | | 1. **Probability of perceiving fair distribution** | |
| --- | --- | --- | --- | --- |
| **Fixed effects^a^** | **Estimate^b^ (SE)** | **Relative importance^c^** | **Estimate^b^ (SE)** | **Relative importance^c^** |
| Intercept | -0.19 (0.13) |  | -5.02 (328.02) |  |
| Compensation (1 = yes, 0 = no) |  |  | 6.97 (583.35) | 1.00 |
| Inside sanctuary (1 = yes, 0 = no) | -0.92 (0.28) | 1.00 | + | 0.25 |
| Household size | 0.20 (0.14) | 0.83 |  |  |
| Fisher association membership (1 = yes, 0 = no) | -0.28 (0.32) | 0.61 | + | 0.29 |
| Food insecurity (1 = insecure, 0 = secure) | 0.14 (0.18) | 0.53 |  |  |
| Household dependency ratio | - | 0.43 |  |  |
| Household income (BDT) | + | 0.40 | - | 0.26 |
| Respondent identity (1 = household head, 0 = other) | + | 0.18 | + | 0.26 |
| Jatka fishing (1 = yes, 0 = no) | - | 0.13 | -0.39 (0.27) | 0.50 |
| Index of fishing dependence | - | 0.13 | - | 0.30 |
| Loan (1 = yes, 0 = no) | + | 0.13 |  |  |
| Awareness (1= high, 0 = low) |  |  | + | 0.15 |
|  |  |  |  |  |
| **# of models in candidate set** | 101 | | 43 | |
| **Random effects^d^** |  |  |  |  |
| Village | 0.21 [0.47] |  | 1.19 [1.09] |  |
| District | 0.00 [0.00] |  | 0.00 [0.00] |  |

Showing the model-averaged coefficient estimates (SE) and relative importance of each variable from the candidate set of models where ΔAICc < 4, based on 642 households from 17 villages in 5 districts.

^a^ Blanks indicate where fixed effects were not included in models.

^b^ Coefficient estimates are presented as contrasts from the intercept, standardised on two standard deviations following Gelman (2008).

^c^ Where the relative importance of a variable is < 0.5, only the direction of the effect is presented.

^d^ Random effects estimates of variance [SD] were taken from the global model.
