# Supplementary material for: Evaluating the ecological and social targeting of a compensation scheme in Bangladesh
Source: PLoS One. 2018 Jun 13;13(6):e0197809. doi: 10.1371/journal.pone.0197809 (PMC5999081; doi:10.1371/journal.pone.0197809)
Supplement: S5 Table — (PDF) [file pone.0197809.s012.pdf]

S5 Table. Model selection table for GLMM with probability of perceiving fair distribution of compensation.

(Intercept)	Compensation	Fishing dependence	Sanctuary	Fisher association membership	Jatka fishing	Respondent identity	Awareness	Household income	df	logLik	AICc	delta	Weight
-11.1137	21.05315	-0.56013	NA	NA	NA	NA	NA	NA	5	-192.404	394.885	0	0.07982
-11.3215	21.65248	-0.56639	1.883684	NA	NA	NA	NA	NA	6	-191.402	394.9103	0.0253	0.078817
-10.9769	20.75321	-0.55416	NA	0.752744	NA	NA	NA	NA	6	-192.133	396.3727	1.487638	0.037938
-10.5378	19.7957	NA	NA	NA	NA	NA	NA	NA	4	-194.174	396.3986	1.513581	0.037449
-11.0471	21.05135	-0.55947	1.869599	0.728506	NA	NA	NA	NA	7	-191.144	396.4312	1.546129	0.036845
-9.7826	18.30999	NA	1.961101	NA	NA	NA	NA	NA	5	-193.205	396.4861	1.601065	0.035846
-11.0635	20.94474	-0.49835	NA	NA	-0.18391	NA	NA	NA	6	-192.246	396.5999	1.714836	0.033864
-11.8661	22.82969	-0.50376	1.850529	NA	-0.18439	NA	NA	NA	7	-191.241	396.626	1.740982	0.033424
-11.0933	21.0055	-0.55521	NA	NA	NA	-0.13674	NA	NA	6	-192.323	396.7541	1.869027	0.031351
-11.2648	21.41709	-0.56255	NA	NA	NA	NA	0.224503	NA	6	-192.349	396.8051	1.920114	0.030561
-10.7439	20.38745	-0.56188	1.879072	NA	NA	-0.12667	NA	NA	7	-191.332	396.8078	1.922823	0.030519
-10.8139	20.39828	-0.56678	NA	NA	NA	NA	NA	-0.03798	6	-192.394	396.8956	2.0106	0.029209
-11.1921	21.37098	-0.57198	1.881488	NA	NA	NA	NA	-0.03242	7	-191.394	396.9314	2.046337	0.028692
-10.9629	20.86687	-0.56642	1.876113	NA	NA	NA	0.010422	NA	7	-191.401	396.946	2.06096	0.028483
-11.4866	21.87166	NA	NA	NA	-0.35901	NA	NA	NA	5	-193.485	397.0471	2.162079	0.027078
-10.2911	19.38283	NA	1.884561	NA	-0.36118	NA	NA	NA	6	-192.503	397.1126	2.227563	0.026206
-11.3265	21.52549	NA	NA	0.810331	NA	NA	NA	NA	5	-193.862	397.8004	2.915356	0.01858
-10.9072	20.77432	NA	1.948964	0.795708	NA	NA	NA	NA	6	-192.901	397.9088	3.0238	0.0176
-11.1314	21.093	-0.49338	NA	0.74602	-0.1811	NA	NA	NA	7	-191.98	398.1025	3.217438	0.015976
-10.9576	20.84031	-0.49821	1.837459	0.71854	-0.18032	NA	NA	NA	8	-190.991	398.1661	3.281115	0.015475
-10.9607	20.71841	NA	NA	NA	NA	-0.15918	NA	NA	5	-194.062	398.2003	3.3153	0.015213
-11.0655	20.94467	-0.54863	NA	0.775601	NA	-0.15032	NA	NA	7	-192.035	398.2134	3.328349	0.015114
-11.1617	21.1959	-0.55722	NA	0.787648	NA	NA	0.270591	NA	7	-192.052	398.2474	3.362361	0.014859
-11.1235	21.21968	-0.55436	1.863764	0.749924	NA	-0.14008	NA	NA	8	-191.06	398.3033	3.418256	0.014449
-10.6672	20.24741	NA	1.953392	NA	NA	-0.14924	NA	NA	6	-193.107	398.3207	3.435675	0.014324
-11.0187	20.87615	NA	NA	NA	NA	NA	0.185871	NA	5	-194.135	398.3465	3.461435	0.014141
-11.7059	22.35313	-0.56192	NA	0.757937	NA	NA	NA	-0.0438	7	-192.119	398.3817	3.496646	0.013894
-11.4158	21.71721	NA	NA	NA	NA	NA	NA	0.039732	5	-194.163	398.4019	3.516919	0.013754
-11.185	21.35592	-0.56616	1.867043	0.733281	NA	NA	NA	-0.03837	8	-191.134	398.4514	3.566358	0.013418
-11.4116	21.85062	-0.55965	1.829683	0.735981	NA	NA	0.055045	NA	8	-191.141	398.4656	3.580614	0.013323

-11.1107	21.04572	-0.49506	NA	NA	-0.18026	-0.13137	NA	NA	7	-192.172	398.4867	3.601631	0.013183
-11.1888	21.38806	NA	1.96272	NA	NA	NA	NA	0.044443	6	-193.191	398.489	3.604016	0.013168
-11.4258	21.73856	NA	NA	0.785168	-0.35424	NA	NA	NA	6	-193.192	398.4921	3.607082	0.013148
-10.5848	20.06766	NA	1.965899	NA	NA	NA	-0.00671	NA	6	-193.205	398.5167	3.631668	0.012987
-11.1108	21.07966	-0.50092	NA	NA	-0.18378	NA	0.224531	NA	7	-192.191	398.5252	3.64013	0.012932
-11.3047	21.60112	-0.5008	1.846617	NA	-0.18102	-0.12121	NA	NA	8	-191.178	398.5405	3.655489	0.012833
-11.1624	21.29374	NA	1.873538	0.76313	-0.35506	NA	NA	NA	7	-192.223	398.5887	3.703627	0.012528
-11.3391	21.55068	-0.50435	NA	NA	-0.18205	NA	NA	-0.03076	7	-192.24	398.6229	3.737857	0.012315
-11.5879	22.22189	-0.50856	1.84907	NA	-0.18285	NA	NA	-0.02489	8	-191.237	398.6585	3.773497	0.012098
-10.4022	19.62223	-0.50379	1.846684	NA	-0.18435	NA	0.00528	NA	8	-191.241	398.6671	3.782028	0.012046
-11.4605	21.84345	-0.55771	NA	NA	NA	-0.13625	0.222482	NA	7	-192.269	398.6814	3.796329	0.011961
-10.6192	19.96829	-0.55958	NA	NA	NA	-0.13269	NA	-0.02431	7	-192.319	398.7819	3.89687	0.011374
-11.2246	21.33157	-0.56986	NA	NA	NA	NA	0.228679	-0.04161	7	-192.337	398.8169	3.931918	0.011177
-11.3036	21.61519	-0.56541	1.877834	NA	NA	-0.12339	NA	-0.01989	8	-191.33	398.8434	3.958426	0.011029
-10.4167	19.67064	-0.56192	1.872766	NA	NA	-0.12664	0.008652	NA	8	-191.332	398.8488	3.963765	0.011
