# Supplementary material for: Evaluating the ecological and social targeting of a compensation scheme in Bangladesh
Source: PLoS One. 2018 Jun 13;13(6):e0197809. doi: 10.1371/journal.pone.0197809 (PMC5999081; doi:10.1371/journal.pone.0197809)
Supplement: S6 Table — (PDF) [file pone.0197809.s013.pdf]

Table S6. Model selection table for GLMM with probability of perceiving fair distribution of compensation, excluding Chandpur.

Intercept	Compensation	Jatka Fishing	Fishing dependence	Fishing association membership	Household income	Respondent identity	Sanctuary	Awareness	df	logLik	AICc	delta	weight
-4.9554	6.8273	-0.4045	NA	NA	NA	NA	NA	NA	5	-103.7503	217.5950	0.0000	0.0810
-5.5301	7.8845	NA	NA	NA	NA	NA	NA	NA	4	-104.8280	217.7189	0.1238	0.0761
-4.9030	6.7714	NA	-0.2214	NA	NA	NA	NA	NA	5	-104.4116	218.9175	1.3225	0.0418
-4.9354	6.8196	NA	NA	0.5163	NA	NA	NA	NA	5	-104.4436	218.9815	1.3865	0.0405
-5.2668	7.3880	-0.4020	NA	NA	-0.1775	NA	NA	NA	6	-103.4711	219.0744	1.4794	0.0386
-4.9646	6.8838	NA	NA	NA	-0.1843	NA	NA	NA	5	-104.5295	219.1534	1.5584	0.0372
-5.0877	7.0661	-0.3667	-0.1492	NA	NA	NA	NA	NA	6	-103.5712	219.2747	1.6797	0.0350
-5.1103	7.0911	-0.4098	NA	NA	NA	0.1851	NA	NA	6	-103.5724	219.2772	1.6822	0.0349
-5.0517	7.0239	NA	NA	NA	NA	0.1747	NA	NA	5	-104.6707	219.4358	1.8407	0.0323
-4.9177	6.7867	-0.3911	NA	0.4878	NA	NA	0.7981	NA	7	-102.7162	219.6090	2.0140	0.0296
-5.3768	7.6121	NA	NA	0.5148	NA	NA	0.8942	NA	6	-103.7972	219.7267	2.1317	0.0279
-4.7513	6.5057	NA	NA	NA	NA	NA	NA	0.0457	5	-104.8209	219.7362	2.1411	0.0278
-4.8265	6.6309	-0.3993	NA	NA	NA	0.1886	0.8155	NA	7	-102.8709	219.9184	2.3234	0.0253
-4.7788	6.5541	-0.3599	-0.1512	NA	NA	NA	0.8077	NA	7	-102.8733	219.9233	2.3283	0.0253
-4.8800	6.7371	NA	-0.2436	NA	-0.2068	NA	NA	NA	6	-104.0362	220.2047	2.6097	0.0220
-4.8263	6.6290	-0.3996	NA	NA	NA	NA	0.8524	-0.0501	7	-103.0478	220.2722	2.6772	0.0212
-5.0667	7.0556	NA	-0.2084	0.4792	NA	NA	NA	NA	6	-104.0760	220.2844	2.6893	0.0211
-4.9989	6.8996	-0.3983	NA	0.5390	-0.1973	NA	NA	NA	7	-103.0583	220.2932	2.6982	0.0210
-4.8518	6.6762	NA	NA	0.5467	-0.2023	NA	NA	NA	6	-104.0964	220.3252	2.7302	0.0207
-5.1642	7.2463	NA	NA	NA	NA	NA	0.9378	-0.0355	6	-104.1750	220.4823	2.8873	0.0191
-4.8312	6.6317	NA	-0.2321	NA	NA	0.1965	NA	NA	6	-104.2164	220.5651	2.9701	0.0183
-4.8697	6.6867	-0.3587	-0.1721	NA	-0.1947	NA	NA	NA	7	-103.2378	220.6523	3.0573	0.0176
-5.1422	7.1527	-0.4083	NA	NA	-0.1962	0.2131	NA	NA	7	-103.2392	220.6551	3.0601	0.0175
-5.2157	7.2669	-0.4053	NA	0.4836	NA	0.1677	NA	NA	7	-103.2443	220.6652	3.0702	0.0174
-5.0480	7.0107	NA	NA	0.4971	NA	0.1564	NA	NA	6	-104.3175	220.7673	3.1723	0.0166
-4.7846	6.5519	NA	NA	NA	-0.2014	0.2024	NA	NA	6	-104.3219	220.7761	3.1811	0.0165
-4.9686	6.8438	-0.4084	NA	0.5363	NA	NA	NA	0.1386	7	-103.3310	220.8388	3.2437	0.0160
-4.8212	6.6370	NA	-0.2268	NA	NA	NA	NA	0.0843	6	-104.3877	220.9077	3.3127	0.0155
-5.0386	6.9661	-0.3691	-0.1579	NA	NA	0.1965	NA	NA	7	-103.3732	220.9230	3.3280	0.0153
-4.9726	6.8968	NA	NA	0.5368	NA	NA	NA	0.0960	6	-104.4124	220.9571	3.3621	0.0151
-4.9323	6.8177	-0.3901	NA	0.5257	-0.2023	NA	0.8165	NA	8	-102.3687	220.9649	3.3699	0.0150
-5.2431	7.3792	NA	-0.2131	0.4734	NA	NA	0.8715	NA	7	-103.4123	221.0013	3.4063	0.0147
-4.8942	6.7705	NA	NA	NA	-0.1922	NA	NA	0.0890	6	-104.5031	221.1384	3.5434	0.0138
-4.7043	6.4259	NA	-0.2374	NA	NA	0.2090	0.8877	NA	7	-103.5183	221.2133	3.6183	0.0133
-5.0621	7.0292	-0.3704	-0.1553	NA	NA	NA	NA	0.1045	7	-103.5368	221.2502	3.6552	0.0130
-4.9905	6.9265	-0.3985	NA	NA	-0.2014	0.2180	0.8352	NA	8	-102.5209	221.2693	3.6742	0.0129
-4.8648	6.6609	-0.4146	NA	NA	NA	0.1856	NA	0.0818	7	-103.5514	221.2795	3.6845	0.0128
-4.8394	6.6683	-0.3527	-0.1742	NA	-0.1990	NA	0.8243	NA	8	-102.5261	221.2798	3.6848	0.0128
-5.0408	7.0088	-0.3562	-0.1387	0.4642	NA	NA	0.7914	NA	8	-102.5623	221.3520	3.7570	0.0124
-4.9885	6.9087	-0.3923	NA	0.4656	NA	0.1711	0.7995	NA	8	-102.5634	221.3542	3.7592	0.0124
-5.1069	7.1337	NA	-0.2328	0.5144	-0.2252	NA	NA	NA	7	-103.6472	221.4710	3.8760	0.0117
-4.8920	6.7496	-0.3578	-0.1599	NA	NA	0.1998	0.8067	NA	8	-102.6675	221.5624	3.9674	0.0111
